# Interplay between microtubule bundling and sorting factors ensures acentriolar spindle stability during *C*. *elegans* oocyte meiosis

**DOI:** 10.1371/journal.pgen.1006986

**Published:** 2017-09-14

**Authors:** Timothy J. Mullen, Sarah M. Wignall

**Affiliations:** Department of Molecular Biosciences, Northwestern University, Evanston, IL, United States of America; Harvard Medical School, UNITED STATES

## Abstract

In many species, oocyte meiosis is carried out in the absence of centrioles. As a result, microtubule organization, spindle assembly, and chromosome segregation proceed by unique mechanisms. Here, we report insights into the principles underlying this specialized form of cell division, through studies of *C*. *elegans* KLP-15 and KLP-16, two highly homologous members of the kinesin-14 family of minus-end-directed kinesins. These proteins localize to the acentriolar oocyte spindle and promote microtubule bundling during spindle assembly; following KLP-15/16 depletion, microtubule bundles form but then collapse into a disorganized array. Surprisingly, despite this defect we found that during anaphase, microtubules are able to reorganize into a bundled array that facilitates chromosome segregation. This phenotype therefore enabled us to identify factors promoting microtubule organization during anaphase, whose contributions are normally undetectable in wild-type worms; we found that SPD-1 (PRC1) bundles microtubules and KLP-18 (kinesin-12) likely sorts those bundles into a functional orientation capable of mediating chromosome segregation. Therefore, our studies have revealed an interplay between distinct mechanisms that together promote spindle formation and chromosome segregation in the absence of structural cues such as centrioles.

## Author summary

When cells divide, they must assemble a microtubule-based structure called a spindle on which the chromosomes are segregated. While in most cell types the microtubules that comprise the spindle are nucleated and organized by centriole-containing centrosomes, female reproductive cells (oocytes) of many species lack centrioles and therefore spindles in these cells assemble using unique mechanisms. Using *C*. *elegans* as a model system, we set out to identify factors required for acentriolar spindle assembly in oocytes and found two microtubule motor proteins necessary for this process. When these motors are depleted in oocytes, microtubules fail to form stable bundles during spindle assembly, resulting in severely aberrant spindles. However, we were surprised to find that these disorganized microtubules were then able to reorganize into a spindle capable of segregating chromosomes during anaphase, revealing a second mechanism that can act to bundle and organize spindle microtubules. Studies of this unique anaphase reorganization phenotype then enabled us to uncover new proteins contributing to spindle organization, and to gain insights into the mechanisms driving chromosome segregation in this important cell type. The work presented here therefore deepens our understanding of the molecular mechanisms of acentriolar spindle assembly and chromosome segregation in oocytes.

## Introduction

During mitosis, centriole-containing centrosomes duplicate and then move to opposite ends of the cell where they nucleate microtubules and form the spindle poles. However, oocytes of many species lack centrioles, and as a result, spindles in these cells assemble using a different pathway [1]. We are interested in understanding the molecular mechanisms underlying this unique, acentriolar pathway of spindle assembly.

Using *C*. *elegans* oocyte meiosis as a model, we recently found that acentriolar spindle assembly in this system proceeds by: 1) formation of a cage-like structure comprised of prominent bundles of microtubules that are constrained by the disassembling nuclear envelope, 2) reorganization of this structure such that the microtubule minus-ends are sorted away from the chromosomes to the periphery of the array where they are focused into multiple nascent poles, and then 3) coalescence of these poles until bipolarity is achieved [2]. During this process, the microtubule bundles project into the space near the homologous chromosome pairs (bivalents) and then begin to form lateral associations with them, an interaction that is maintained through anaphase. These lateral associations contribute to the alignment of bivalents at metaphase [3]. Subsequently, during anaphase, spindle morphology changes: the spindle shrinks and rotates 90 degrees such that it is perpendicular to the cell cortex, the spindle poles broaden, and the microtubule bundles reorganize into a parallel array, creating open channels [4–6]. Anaphase then proceeds through two phases, with chromosome-to-pole movement through the open channels in Anaphase A, and spindle elongation driving chromosomes further apart in Anaphase B [7]. This unique mode of chromosome segregation is kinetochore-independent [8], and instead relies on a complex of proteins containing AIR-2 (Aurora B kinase) that concentrates at the center of each bivalent [9, 10], forming a ring-like structure (the “midbivalent ring”). These rings localize to chromosomes during spindle formation [3] and then are removed from chromosomes in anaphase, remaining in the channels in the center of the spindle [8].

In this system, numerous factors have been shown to contribute to different aspects of acentriolar spindle assembly (e.g., microtubule length regulation and spindle pole formation), including MEI-1/2 (katanin), KLP-7 (MCAK), ASPM-1, dynein, and others (reviewed in [11]). Moreover, the kinesin-12 family member KLP-18 promotes spindle bipolarity [3, 12, 13], by sorting microtubule bundles and forcing the minus-ends outward where they can be organized into the spindle poles [2]. However, the factors that are required for bundling microtubules and stabilizing these bundles in the absence of centrioles are unknown. Furthermore, little is known about how the acentriolar anaphase spindle is organized and stabilized. During mitosis in *C*. *elegans*, the centralspindlin complex of CYK-4 (RhoGAP) and the kinesin-6 family member ZEN-4 (MKLP1) binds to and bundles antiparallel microtubules in the midzone of the anaphase spindle, providing stability to the structure [14]. The centralspindlin complex also localizes to the meiotic anaphase spindle in oocytes, and although this complex is required for the completion of cytokinesis and polar body formation [15], depletion has no effect on anaphase spindle morphology [8]. Another component important for anaphase spindle organization during *C*. *elegans* mitosis is the microtubule bundling protein SPD-1 (PRC1), which is required for proper central spindle structure [16–18], and localizes to the midzone in mitosis [16] and meiosis [19, 20]. However, depletion of SPD-1 from *C*. *elegans* oocytes does not produce an obvious phenotype [8], making it unclear if this protein functions during oocyte meiosis.

Now, we have identified KLP-15 and KLP-16, members of the conserved kinesin-14 family of minus-end-directed kinesins [21], as factors required for microtubule bundling and organization during acentriolar spindle assembly in *C*. *elegans* oocytes; in the absence of these proteins, spindles are unable to maintain stable microtubule bundles and as a result are severely aberrant at metaphase and early anaphase. However, despite these defects, microtubules are then able to reorganize into a spindle capable of mediating chromosome segregation during anaphase. Importantly, this unexpected spindle reorganization phenotype enabled us to gain new insights into the mechanisms underlying anaphase spindle organization and chromosome segregation during acentriolar meiosis, uncovering previously unidentified roles for SPD-1 and KLP-18 in anaphase. These studies have therefore revealed a role for minus-end kinesins in acentriolar spindle assembly in *C*. *elegans* oocytes and highlight how the interplay of multiple mechanisms functions to promote the formation of a bipolar spindle that is capable of faithfully segregating chromosomes in this specialized type of cell division.

## Results

### KLP-15 and KLP-16 are required for acentriolar spindle assembly in oocytes

**Fig 1:**
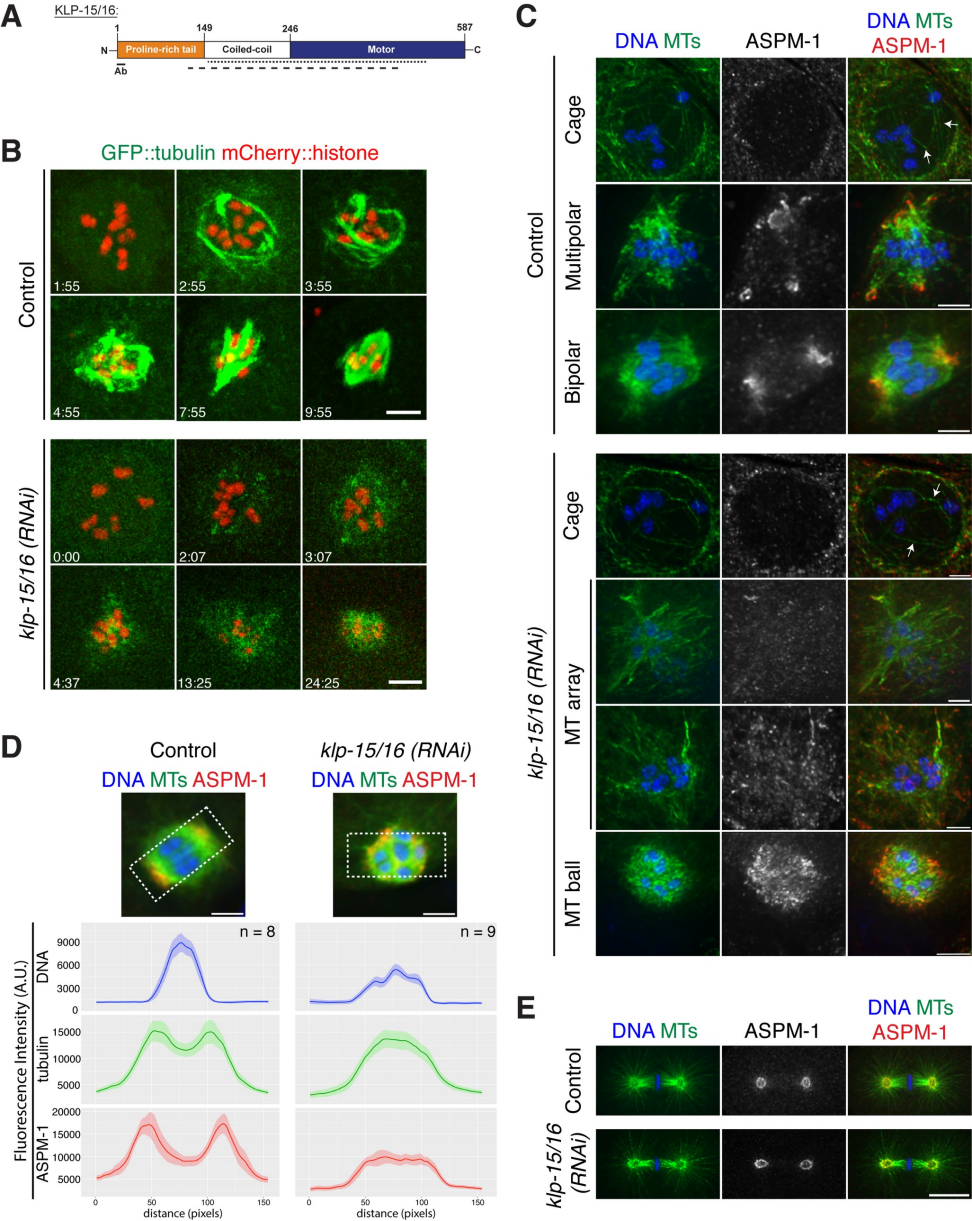
Depletion of KLP-15/16 results in disorganized oocyte spindles. (A) Schematic of KLP-15/16 with the proline-rich tail in orange, coiled-coil domain in white, and the motor domain in blue. The dotted line represents where *klp-15(RNAi)* targets, and the dashed line represents where *klp-16(RNAi)* targets. The underlined region labeled “Ab” is the region of the protein our antibody was made against. (B) Movie stills from control and *klp-15/16(RNAi)* oocytes expressing GFP::tubulin and mCherry::histone. In the control, after NEBD, diffuse tubulin can be seen inside the nucleus as was recently reported [20]. Then microtubules are nucleated and bundled to form a cage-like structure before reorganizing into a multipolar spindle and achieving bipolarity (in 5/5 control movies examined, spindles achieved bipolarity prior to anaphase onset). Following *klp-15/16(RNAi)*, microtubules form a transient cage that then forms a disorganized array that fails to achieve bipolarity and collapses into a microtubule ball (in 6/6 *klp-15/16 (RNAi)* movies examined, spindles entered anaphase without achieving bipolarity); although strong cage bundles are not evident in this particular example, fixed imaging has confirmed that they are able to form. (C, D, and E) Meiotic (C and D) and mitotic (E) spindles stained for DNA (blue), tubulin (green) and ASPM-1 (red). (C) In the control, ASPM-1 marks spindle poles at the multipolar and bipolar stages. In *klp-15/16(RNAi)* oocytes, the microtubule cage forms, but then aberrant structures form with diffuse ASPM-1 staining. (D) Line scans of metaphase spindles in control and *klp-15/16(RNAi)* oocytes. 6 z-slice sum projections of spindles were used for the analysis and the average fluorescence intensities are graphed (solid line) with the SEM (shaded area). The y-axes of the graphs are the same for the control and experimental conditions for a given channel. (E) Mitotic spindles are normal in *klp-15/16(RNAi)* embryos (12/12 *klp-15/16(RNAi)* spindles analyzed were bipolar and indistinguishable from control spindles). Bars = (B) 5 μm; (C and D) 2.5 μm; (E) 10 μm.

To identify proteins required for acentriolar spindle assembly in *C*. *elegans* oocytes, we performed a targeted RNAi screen of genes previously reported to be embryonic lethal, visually screening for spindle defects in a strain expressing GFP::tubulin and GFP::histone [3]. This screen identified KLP-15 and KLP-16, two highly homologous minus-end-directed kinesins (91.1% identical in amino acid sequence and 93% identical in mRNA sequence) of the kinesin-14 family [22]. We observed the same spindle defects when we used the RNAi library clone annotated as targeting *klp-15* as we did when we used the *klp-16* clone, likely because both RNAi constructs target both transcripts due to the high sequence similarity between them (Figs 1A and S1). Consistent with this interpretation, our RNAi conditions caused high embryonic lethality (95.6%; S2A Fig), whereas single deletion mutants of either motor were largely viable; *klp-15(ok1958)* had 14% embryonic lethality and a new deletion mutant we generated, *klp-16(wig1)*, had 2.6% embryonic lethality. Moreover, treatment of *klp-15(ok1958)* with the RNAi clone annotated as targeting *klp-15* and treatment of *klp-16(wig1)* with the clone annotated as targeting *klp-16* caused high embryonic lethality (89.8% and 94.6%, respectively; S2A Fig), consistent with the interpretation that both proteins are expressed, and that each RNAi library clone can target both proteins. These results suggest that KLP-15 and KLP-16 are redundant, and therefore, we refer to these proteins collectively as KLP-15/16 (in describing assays and results where we cannot distinguish between them).

Previous work from other groups had suggested a role for KLP-15/16 in the segregation of meiotic chromosomes, because in addition to embryonic lethality, inhibition of these proteins resulted in phenotypes such as polar body defects, a high incidence of male progeny (which results from non-disjunction of the X chromosome in the oocyte) and multiple female pronuclei in the one-cell stage embryo [21, 23–28]. However, a careful analysis to determine the causes of these segregation errors had not been reported. Therefore, we performed detailed live and fixed imaging of oocytes following *klp-15/16(RNAi)*. After nuclear envelope breakdown (NEBD) is initiated in control oocytes, microtubules form prominent bundles that organize into a cage-like structure (Fig 1B, Fig 1C, arrows; S1 Movie). The microtubules are then sorted such that the minus-ends of the microtubule bundles, visualized by ASPM-1 [29–31], are on the periphery of the array, where they are organized into multiple nascent poles that coalesce until bipolarity is achieved (Figs 1B, 1C, 1D and S2C; S1 and S3 Movies [2]). In *klp-15/16(RNAi)* oocytes, although the microtubule cage appears to initially form normally (Fig 1C bottom; arrows), the microtubule bundles are not maintained and begin to fall apart, resulting in a disorganized array that lacks focused nascent poles. Then, the array collapses into a “microtubule ball” comprised of short microtubules surrounding the chromosomes (Figs 1B, 1C and S2C; S1 and S2 Movies). ASPM-1 localization appears largely diffuse at both the microtubule “array” and “ball” stages (Figs 1C, 1D and S3A; S4 Movie), suggesting that microtubule minus-ends are distributed throughout these structures. However, since there are examples of spindles where ASPM-1 does have areas of slight concentration within the microtubule ball structures (S3A Fig, arrowheads), it is possible that these spindles have some small degree of microtubule organization.

**Fig 2:**
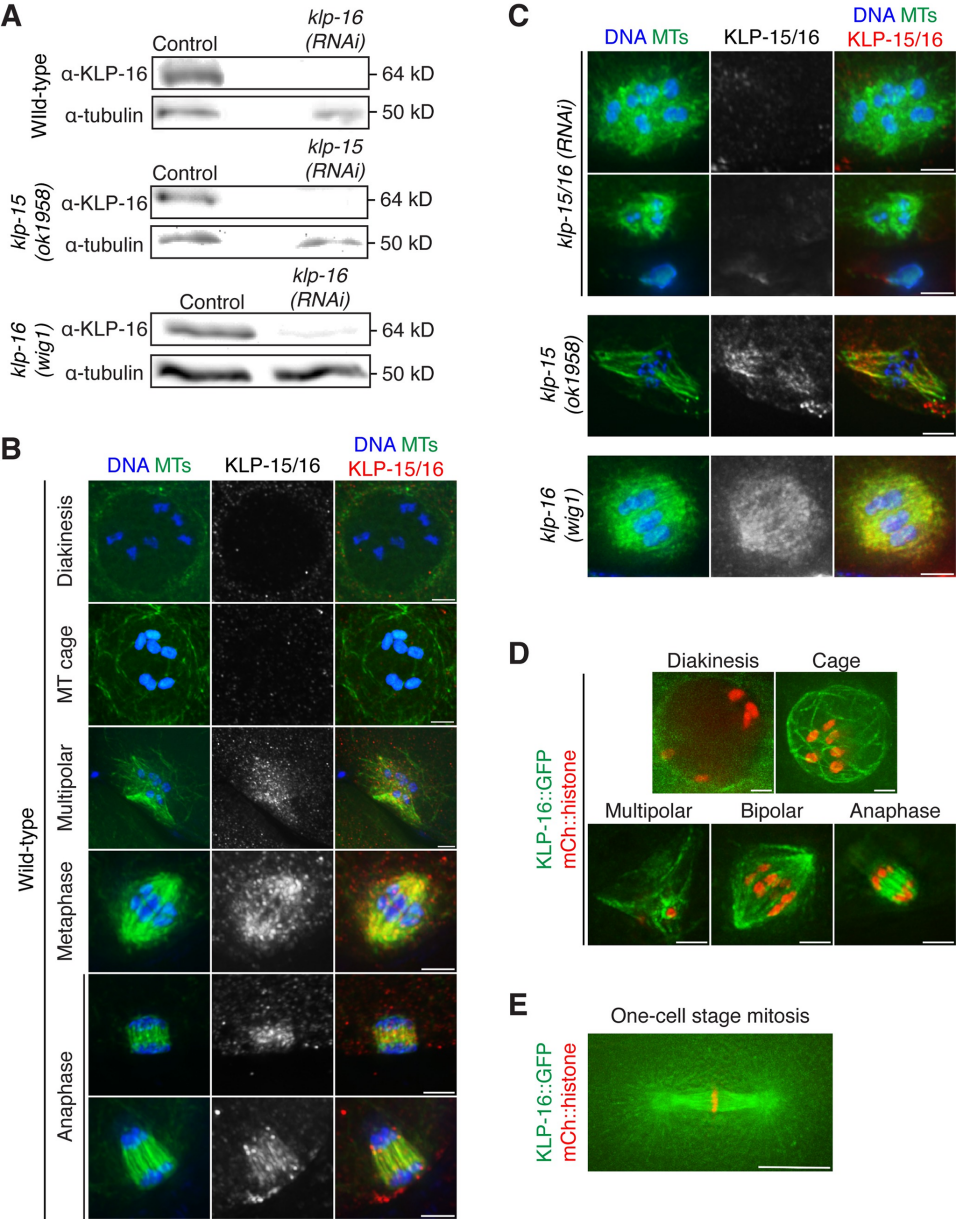
KLP-15/16 localize to spindle microtubules during oocyte meiosis. (A) Western blot from control, *klp-15 or klp-16(RNAi)* worms probed with the KLP-16 antibody or tubulin as a loading control. (B and C) DNA (blue), tubulin (green) and KLP-15/16 (red). (B) Wild-type meiotic spindles at all stages of spindle assembly; KLP-15/16 begin to accumulate on microtubules at the multipolar stage and remain associated through anaphase (quantification in Materials and Methods). (C) The KLP-15/16 signal is lost following *klp-15/16(RNAi)*, though staining of *klp-15(ok1958)* and *klp-16(wig1)* is not different from wild-type spindles (quantification in Materials and Methods). (D and E) Live imaging of worms expressing KLP-16::GFP and mCherry::histone shows that KLP-16 localizes to microtubule bundles at the cage stage (which was not apparent in the KLP-16 antibody staining) and remains associated with the spindle (D). The multipolar image is a partial projection of the entire structure. Stages of spindle assembly were discerned by spindle morphology, chromosome organization, and position in the germline. (E) KLP-16 is also present on mitotic spindle microtubules and centrosomes at the one-cell stage (in 5/5 embryos analyzed). Bars = (B, C, and D) 2.5 μm; (E) 10 μm.

Our analysis therefore demonstrates that depletion of KLP-15/16 affects the early stages of spindle assembly, resulting in structures that lack prominent microtubule bundles past the cage stage. These two kinesins likely function redundantly at this stage, since spindles appeared normal in the *klp-15(ok1958)* and the *klp-16(wig1)* single mutants (Figs 2C and S3C). Interestingly, despite the severe spindle defects in oocytes following *klp-15/16(RNAi)*, we observed that mitotic spindles in the one-cell stage embryo formed normally (Fig 1E; S5 Movie), suggesting that these proteins are not essential when centriole-containing centrosomes are present.

### KLP-15/16 localize to spindle microtubules throughout oocyte meiosis

Given the strong phenotype observed in oocytes upon KLP-15/16 depletion, we next assessed the localization of these proteins. To this end, we generated a peptide antibody against the N-terminal 20 amino acids of KLP-16; because there is only one amino acid different between KLP-15 and KLP-16 in this region, this antibody likely recognizes both proteins (S1 Fig). Indeed, this antibody recognizes a band corresponding to the size of both proteins in a Western blot of control worms, and this band was greatly reduced when RNAi was performed using a clone from the RNAi library that had been annotated as targeting *klp-16* (Fig 2A). Furthermore, this band was also reduced when *klp-15(ok1958)* worms were treated with the clone annotated as targeting *klp-15*, and when *klp-16(wig1)* worms were treated with the *klp-16* clone (Fig 2A), further demonstrating the specificity of the antibody and confirming that RNAi treatment using either of the RNAi library clones for *klp-15* or *klp-16* targets both proteins.

Using this antibody, we found that KLP-15/16 localize in the cytoplasm prior to NEBD and then begin to accumulate on microtubules during the multipolar stage, becoming uniform on the spindle throughout metaphase and anaphase (Fig 2B). This localization is specific and likely represents both proteins, as it is abolished following RNAi depletion of KLP-15/16, but it is the same as wild-type in the *klp-15(ok1958)* mutant and the *klp-16(wig1)* mutant (Fig 2C). We observed a similar localization pattern in a worm strain expressing KLP-16::GFP from the endogenous locus, but this strain also revealed clear localization of KLP-16 to microtubule bundles at the cage stage (Figs 2D and S4B) and to the centrosomes and mitotic spindle microtubules in one-cell stage embryos (Figs 2E and S4C), patterns that were not apparent with the KLP-15/16 antibody (quantification in Materials and Methods). This discrepancy is likely due to variability with the fixed imaging, because even for the stages where we could observe robust staining of spindle microtubules using the KLP-15/16 antibody, not all spindles were stained. Furthermore, when we stained oocytes from the KLP-16::GFP strain with a GFP antibody, we saw similar variability (quantification in Materials and Methods) (S4D and S4E Fig), despite the fact that when this strain was viewed live, every oocyte/embryo imaged had bright KLP-16 fluorescence that appeared to mark spindle structures (S4A Fig). Therefore, we conclude that KLP-15/16 localize to spindle structures through all stages of oocyte spindle assembly, and also to microtubules in mitotic one-cell stage embryos.

Although KLP-15/16 localize to microtubule bundles at the cage stage (Figs 2D and S4B), these motors are not necessary for the formation of these bundles (Fig 1C), suggesting that they act redundantly with other microtubule associated factors at this initial stage of spindle assembly. Similarly, KLP-15/16 localize to spindle microtubules in mitotic embryos (Figs 2E, S4C and S4E), but they are not necessary for the assembly of these spindles (Fig 1E), potentially because centrosomes provide the primary source of microtubule organization in these cells. Taken together, the phenotype of *klp-15/16(RNAi)* and the localization pattern of these proteins support a role for KLP-15/16 in acentriolar meiotic spindle assembly where they likely stabilize the microtubule bundles formed during the cage stage. These stabilized microtubule bundles can then be sorted by other molecular motors such as KLP-18 to achieve bipolarity [2].

### Microtubules bundle and chromosomes segregate in anaphase following KLP-15/16 depletion

While filming *klp-15/16(RNAi)* oocytes, we made the surprising observation that although spindle assembly was severely aberrant, microtubules were often able to reorganize into a bundled structure capable of segregating chromosomes, suggesting the presence of a second, KLP-15/16-independent mechanism for bundling microtubules that operates during anaphase (S6 Movie). Therefore, we used markers of anaphase progression to carefully examine anaphase in *klp-15/16(RNAi)* oocytes, to better understand this mechanism.

**Fig 3:**
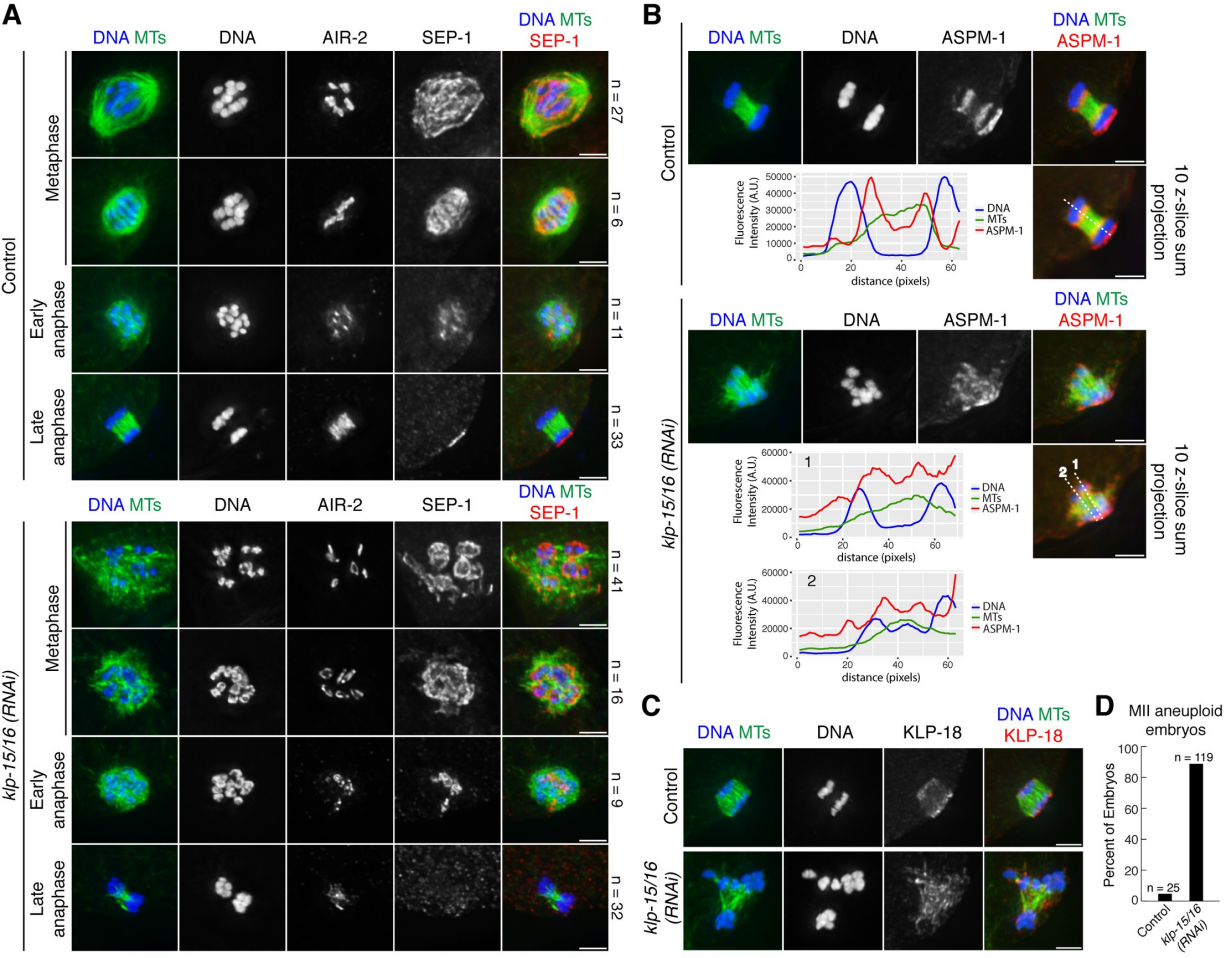
Microtubules bundle and chromosomes segregate in anaphase in *klp-15/16(RNAi)* oocytes. (A) DNA (blue), tubulin (green), AIR-2 (not in merge) and SEP-1 (red in merge), showing anaphase progression in control and *klp-15/16(RNAi)* oocytes. At metaphase, AIR-2 is in the midbivalent ring and SEP-1 displays a staining pattern characteristic of outer kinetochore proteins in *C*. *elegans* oocytes (cupping the bivalents and also forming filamentous linear structures within the spindle [61]). In early anaphase, SEP-1 co-localizes with AIR-2 in the rings, and in late anaphase, SEP-1 is gone and AIR-2 is relocalized onto the microtubules. In *klp-15/16(RNAi)* oocytes, microtubules are disorganized in metaphase and early anaphase, but in late anaphase microtubules are bundled and chromosomes segregate into distinct masses; note that SEP-1 also localizes to the cell cortex in late anaphase. n represents the number of spindles observed for each condition. (B and C) Anaphase spindles with DNA (blue), tubulin (green), and ASPM-1 (B) or KLP-18 (C) (red). (B) Line scans of anaphase spindles in control and *klp-15/16(RNAi)* oocytes. The top row for each condition is a max projection of the entire spindle and the image used for the line scan is a 10 z-slice sum projection. ASPM-1 is enriched at the poles in control spindles (21/24 of spindles analyzed had ASPM-1 enriched at two poles; 88%), but is diffuse along spindle microtubules in anaphase of *klp-15/16(RNAi)* oocytes (only 9/26 spindles could be classified as having any type of ASPM-1 enrichment at pole-like foci; 35%); in both cases ASPM-1 also displays cortical localization. (C) KLP-18 is enriched at spindle poles in the control (11/12 spindles analyzed; 92%), but is localized throughout the spindles in *klp-15/16(RNAi)* oocytes (6/6 spindles had diffuse KLP-18 localization). (D) Percentage of aneuploid MII embryos from control and *klp-15/16(RNAi)* worms. Chromosomes were counted in images of MII embryos, and embryos were counted as aneuploid if the number of chromosomes did not equal 6. Bars = 2.5 μm.

During meiosis in wild-type oocytes, separase (SEP-1) relocates from the kinetochore to the midbivalent ring complex at anaphase onset and then disappears from the rings by late anaphase (Fig 3A) [4]. Aurora B (AIR-2), a component of the midbivalent ring complex, is removed from the rings at anaphase onset and relocalizes to the microtubules by mid anaphase (Fig 3A) [32, 33]. Therefore, we used these markers to stage oocytes following *klp-15/16(RNAi)*, allowing us to distinguish pre-anaphase (AIR-2 on the ring structures, SEP-1 on kinetochore), early anaphase (both proteins in rings), and mid/late anaphase (AIR-2 on microtubules, SEP-1 gone).

Using these markers to stage *klp-15/16(RNAi)* spindles, we found that the microtubule ball configuration observed in our imaging (Fig 1B, 1C and 1D) represents a mixture of metaphase and early anaphase (Fig 3A), although the structures in early anaphase tended to be smaller (S3B Fig). (Note that the spindles that we used for our linescan analysis in Fig 1D all were within the range of volumes observed for metaphase spindles (S3B Fig)). This analysis suggests that the metaphase disorganized microtubule array begins to shrink in preparation for anaphase, similar to what happens in wild-type spindles [6]. Following this stage, when AIR-2 has relocalized to the microtubules and SEP-1 is gone in mid/late anaphase, microtubules reform into a bundled structure and chromosomes are able to segregate into distinct masses (Fig 3A). Despite this anaphase spindle reorganization, we observed segregation errors such as lagging chromosomes and segregation of chromosomes along different axes (Fig 3B and 3C; S6 Movie) that resulted in high levels of aneuploidy in MII oocytes (Fig 3D), likely due to the severely aberrant metaphase spindles that were unable to align chromosomes (Fig 1B and 1C). However, the high rates of aneuploidy also raised the possibility that the microtubules in the *klp-15/16(RNAi)* anaphase spindles may not be organized like in wild-type spindles, where a high concentration of microtubule minus-ends are found at the spindle poles. To test this hypothesis, we assessed the localization of ASPM-1 and KLP-18 (a kinesin that is enriched at the poles of wild-type oocyte spindles [12]), and found that the microtubules of anaphase spindles in *klp-15/16(RNAi)* oocytes, although bundled, are likely not organized properly, since ASPM-1 and KLP-18 are distributed throughout the entire spindle instead of being enriched at the poles (Fig 3B and 3C). Although it is possible that microtubules within the bundles are properly organized and that the signals to localize ASPM-1 and KLP-18 are defective, we favor the interpretation that the secondary mechanism we identified bundles microtubules without first sorting them, resulting in bundles comprised of microtubules of mixed polarity.

### KLP-15/16-independent anaphase microtubule bundling is mediated by SPD-1

**Fig 4:**
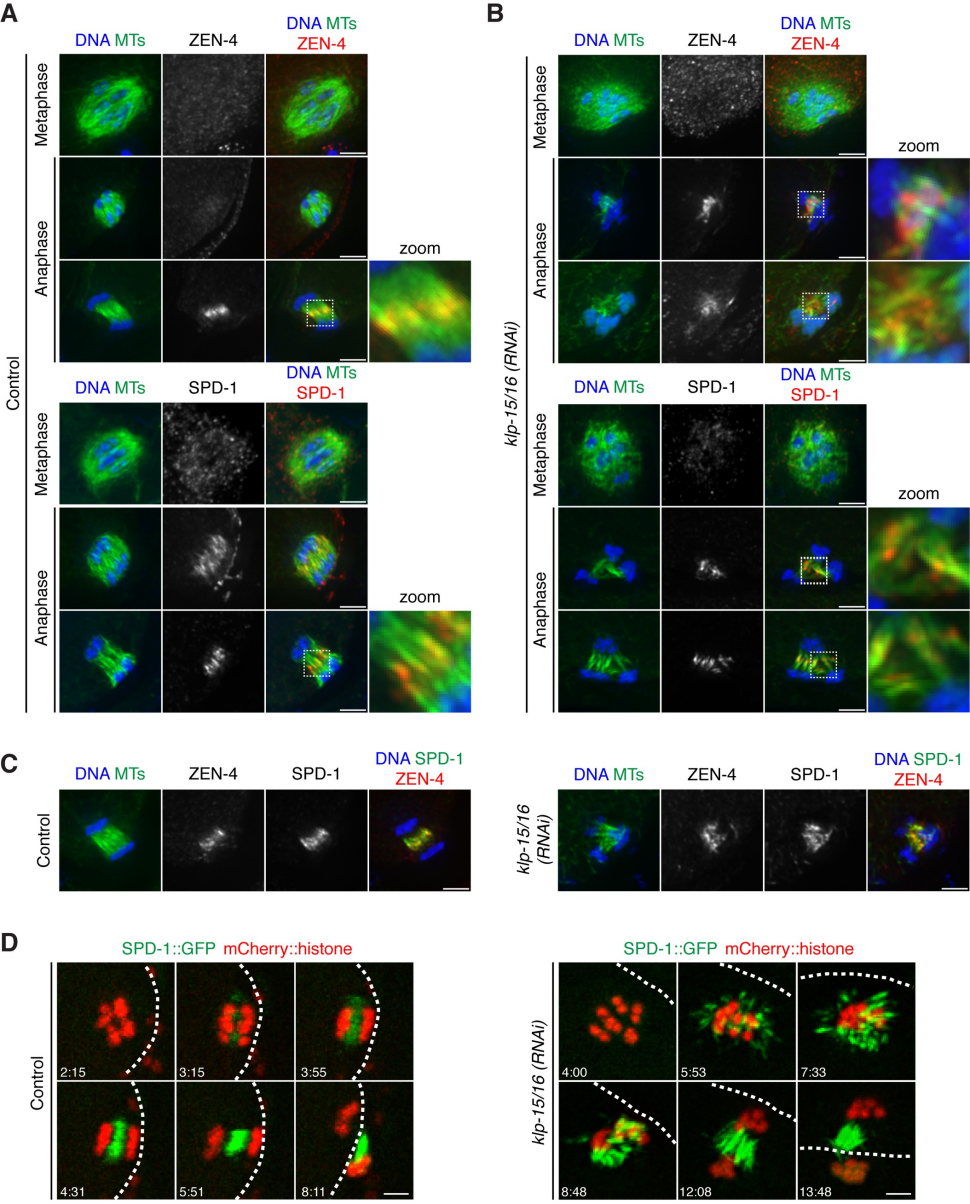
SPD-1 and centralspindlin localize to anaphase spindle microtubules. (A and B) DNA (blue), tubulin (green) and ZEN-4 (top) or SPD-1 (bottom) (red) in control (A) or *klp-15/16(RNAi)* (B) oocytes. ZEN-4 does not localize to metaphase spindles in either case. In anaphase, ZEN-4 localizes to a distinct region in the midzone of control spindles (A, top zoom), but is sometimes not seen on spindles with a short chromosome segregation distance. Following *klp-15/16(RNAi)*, ZEN-4 localizes to the microtubule bundles but is not concentrated to as distinct a band as in the control (B, top zoom). In both control and *klp-15/16(RNAi)* oocytes, SPD-1 does not localize to metaphase spindles. In control oocytes, SPD-1 localizes to the microtubules between the chromosomes in early anaphase and then becomes concentrated in a band on the microtubule bundles in the center of the spindle (A, bottom zoom). Following *klp-15/16(RNAi)*, SPD-1 localizes to microtubule bundles between segregating chromosomes, even when bundles are not all oriented along the same axis (B, bottom zoom) (quantification in Materials and Methods). (C) DNA (blue), tubulin (green), ZEN-4 (red) and SPD-1 (green in final merge). In both control and *klp-15/16(RNAi)* oocytes, ZEN-4 and SPD-1 have similar but distinct localization patterns. (D) Movie stills of oocytes expressing mCherry::histone and SPD-1::GFP in both control and *klp-15/16(RNAi)*. In control spindles, SPD-1 localizes between chromosomes as they begin to segregate and continues to accumulate as anaphase progresses, consistent with previous studies [19, 20]. Following *klp-15/16(RNAi)*, SPD-1 begins to load all over the microtubules, and as SPD-1 accumulates, long bundles begin to form that then orient along the same axis and the chromosomes segregate (4/4 movies analyzed). White dashed lines = cell cortex. Bars = 2.5 μm.

Next, we wanted to uncover factors that are responsible for bundling anaphase microtubules in *klp-15/16(RNAi)* oocytes. Two possible candidates are the centralspindlin complex (comprised of ZEN-4 and CYK-4) and SPD-1, since these proteins have clearly defined roles during anaphase in *C*. *elegans* mitosis [14, 16, 18, 34] and have been shown to concentrate at the midzone of the anaphase spindle in *C*. *elegans* oocytes [8, 19, 20]. Therefore, we assessed the localization of SPD-1 and ZEN-4 at high resolution on *C*. *elegans* oocyte spindles. As expected from previous studies, we found that neither centralspindlin (ZEN-4) nor SPD-1 localize to metaphase spindles in control oocytes (Fig 4A). However, during anaphase, ZEN-4 and SPD-1 both become enriched in a short region at the center of the spindle (Fig 4A), with similar though non-identical localization (Fig 4C). Following *klp-15/16(RNAi)*, we observed a similar pattern, with neither ZEN-4 nor SPD-1 present on the disorganized spindle structures prior to anaphase, but then prominent localization on the bundled microtubules between the sets of segregating chromosomes during anaphase (Fig 4B). This localization was clear even in spindles with multiple sets of segregating chromosomes, where the bundles were not all oriented along the same axis (Fig 4B, bottom zoom). Therefore, because centralspindlin and SPD-1 both localize to microtubule bundles following *klp-15/16(RNAi)*, these factors are in a location where they could potentially contribute to the anaphase-bundling mechanism we identified.

**Fig 5:**
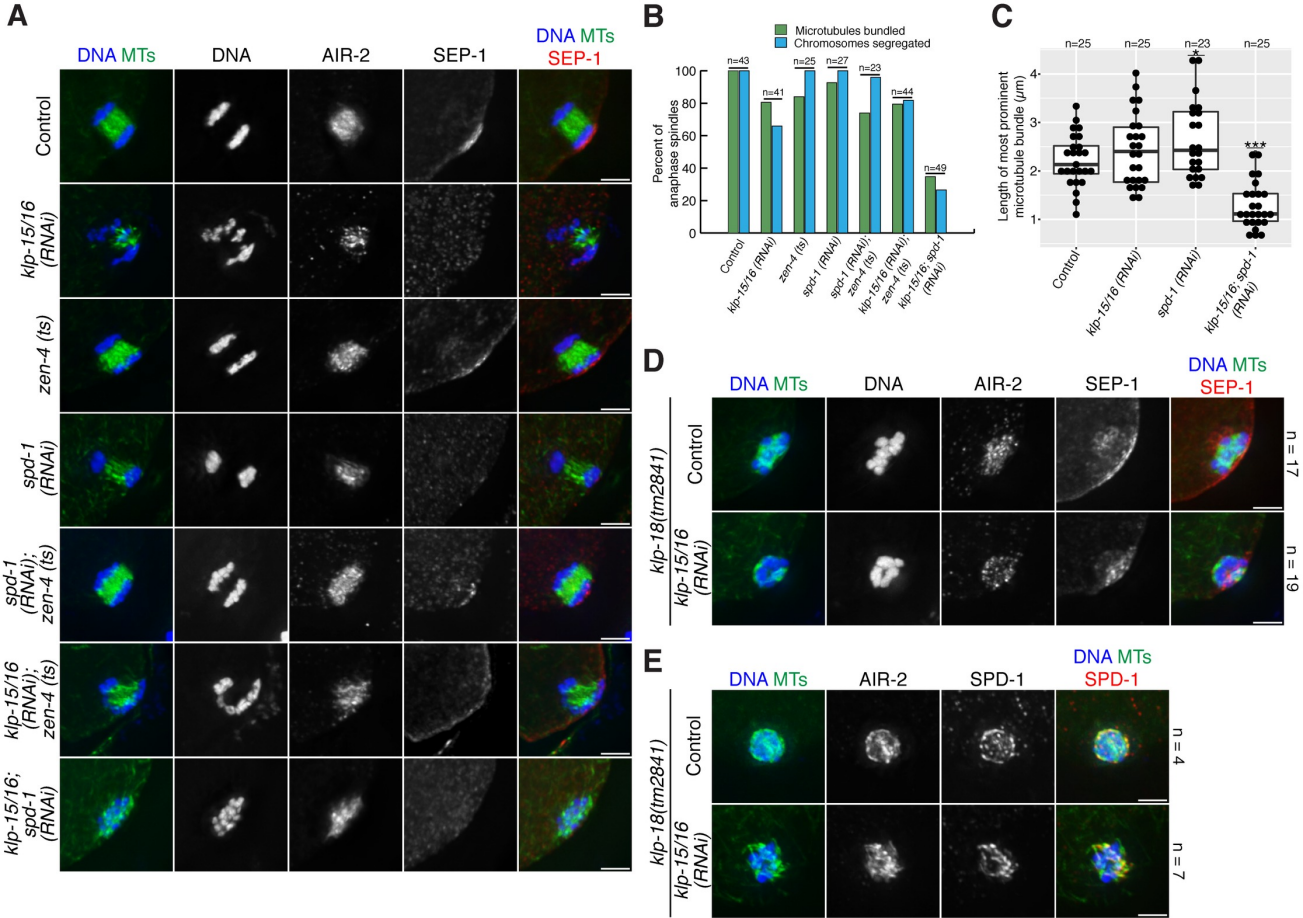
SPD-1 and KLP-18 are required for KLP-15/16-independent spindle reorganization during anaphase. (A and D) DNA (blue), tubulin (green), AIR-2 (not in merge) and SEP-1 (red in merge); all spindles shown are mid/late anaphase, with SEP-1 gone and AIR-2 relocalized to the microtubules. (A) Singly depleting/inhibiting *klp-15/16*, *spd-1*, or *zen-4* or doubly depleting/inhibiting *klp-15/16;zen-4* and *spd-1;zen-4* all resulted in anaphase spindles that were able to bundle microtubules and segregate chromosomes, while double depletion of *klp-15/16;spd-1* abolished microtubule bundling and chromosome segregation. (B) Quantification of the experiment shown in (A). The simple matching coefficient for microtubule bundling and chromosome segregation = 0.82 (n = 251); in other words, 82% of the spindles either showed chromosome segregation when microtubules were bundled or did not show chromosome segregation when microtubules were not bundled. (C) Box plots showing anaphase microtubule length measurements; for a given image, the most prominent and longest microtubule bundle in the spindle was measured. Box represents first quartile, median, and third quartile. Lines extend to data points within 1.5 interquartile range. Asterisks (***) represent significant difference (p < 0.001, two tailed t-test) compared to the other three conditions; (*) represents significant difference (p < 0.05, two tailed t-test) compared to control conditions. (D) Anaphase spindle reorganization and chromosome segregation are not observed in *klp-18(tm2841)* following either control or *klp-15/16* RNAi; in both conditions microtubules are disorganized and segregation fails, suggesting that KLP-18 could potentially mediate the anaphase spindle reorganization observed in KLP-15/16-depleted oocytes. n represents the number of spindles observed for each condition. (E) DNA (blue), tubulin (green), AIR-2 (not in merge) and SPD-1 (red in merge); SPD-1 localizes to spindle microtubule bundles in *klp-18(tm2841)* and *klp-18(tm2841);klp-15/16 (RNAi)* oocytes. n represents the number of spindles observed for each condition. Bars = 2.5 μm.

To test this hypothesis, we assessed a potential functional role for these proteins in anaphase microtubule bundling. In previous work, co-depletion of ZEN-4 and SPD-1 did not affect anaphase spindle structure [8], suggesting that these proteins may not play a role in oocytes. However, our studies have revealed a mechanism that operates in parallel with KLP-15/16 (since KLP-15/16 are normally present on anaphase microtubules, Fig 2B and 2D). Thus, we expect that single depletion of this putative anaphase bundling factor may have only a mild (or no) anaphase phenotype, but that depletion of KLP-15/16 in combination with the secondary factor would completely abolish anaphase bundling. Therefore, we tested each candidate by single depletion and also by co-depletion/inhibition with KLP-15/16 (Fig 5A), and then scored microtubule bundling in mid/late anaphase (using SEP-1 and AIR-2 as markers to stage anaphase, as before; Fig 5B). In addition to microtubule bundling, we also assessed chromosome segregation as a functional readout for anaphase spindle organization, by scoring whether chromosomes were able to segregate into distinct masses (Fig 5B).

**Fig 6:**
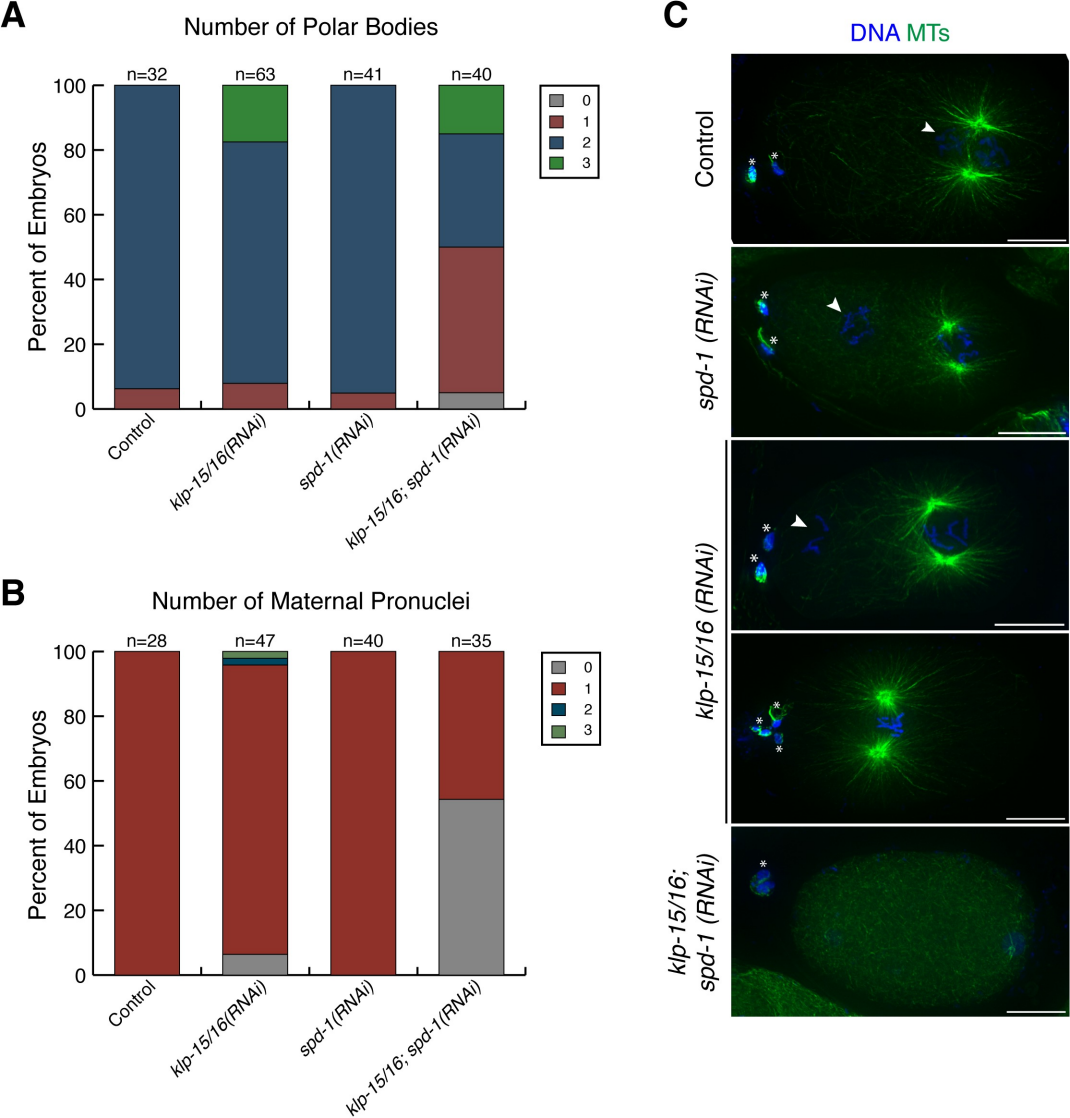
Consequences of the anaphase defects observed in *klp-15/16(RNAi)* oocytes. (A and B) Analysis of polar bodies and maternal pronuclei was done using live worms expressing GFP::tubulin, GFP::histone. (A) Quantification of the number of polar bodies per embryo for each condition listed. (B) Quantification of the number of maternal pronuclei per embryo for each condition listed. For A and B, embryos were only scored if the paternal pronucleus was decondensed to ensure that the meiotic divisions were complete. (C) Example mitotic embryos showing DNA (blue) and tubulin (green) to show some of the phenotypes observed in the quantification displayed in A and B. Asterisks denote polar bodies and arrowheads denote maternal pronuclei. We observe extra polar bodies and pronuclei following *klp-15/16(RNAi)*, indicative of meiotic defects. Moreover, co-depletion of KLP-15/16 and SPD-1 often results in ejection of all maternal chromosomes into a single polar body, resulting in no maternal pronucleus. Bars = 10 μm.

Using these assays, we found that both single and double inhibition/depletion of ZEN-4 and SPD-1 had little effect on anaphase microtubule bundling and chromosome segregation (Fig 5A and 5B), consistent with a previous study [8]. Moreover, oocytes where both ZEN-4 and KLP-15/16 were inhibited/depleted appeared similar to *klp-15/16(RNAi)* alone, with most spindles containing bundled microtubules that were able to segregate chromosomes. However, we found that co-depletion of KLP-15/16 and SPD-1 largely abolished anaphase microtubule bundling and chromosome segregation (Fig 5A and 5B) and resulted in spindles with shorter microtubule lengths (Fig 5C). Furthermore, we observed an increase in the percentage of embryos with a single large polar body and no maternal pronucleus under these conditions, suggesting that the meiotic divisions lacked a functional spindle on which DNA could segregate (Fig 6A, 6B and 6C). Interestingly, our estimations of spindle microtubule lengths revealed that the microtubules in the *spd-1(RNAi)* condition were somewhat longer than microtubules in the control (Fig 5C), suggesting that SPD-1 may perform a subtle role in regulating spindle length in anaphase. This observation is reminiscent of studies of mitotic anaphase in *C*. *elegans*, where an SPD-1 mutant displays larger distances between segregating chromosomes than wild-type embryos, suggesting that the microtubule crosslinking activity of SPD-1 can act to slow the rate of spindle midzone elongation [35]. Taken together, these data highlight a previously unknown role for SPD-1 on acentriolar anaphase spindles.

Given this finding, we more carefully assessed the dynamics of SPD-1 loading onto the spindle during anaphase. Live imaging of control oocytes expressing SPD-1::GFP and mCherry::histone revealed that SPD-1 begins to load onto the spindle between segregating chromosomes shortly after spindle rotation and then continues to accumulate as anaphase progresses (Fig 4D; S7 Movie), consistent with previous studies [19, 20]. Similar to control oocytes, following depletion of KLP-15/16, SPD-1 loads onto microtubules in early anaphase, at the microtubule ball stage (Fig 4D; S7 Movie). Subsequently, as SPD-1 accumulates on the spindle, prominent bundles begin to form (Fig 4D; S7 Movie). This localization pattern, in combination with our functional analysis, is consistent with the interpretation that loading of SPD-1 in early anaphase provides a secondary bundling activity that provides spindle stability and allows for chromosome segregation.

### KLP-18 potentially facilitates the reorganization of microtubule bundles during KLP-15/16-independent anaphase

Our SPD-1::GFP imaging also revealed that when SPD-1 loads onto microtubules in *klp-15/16(RNAi)* oocytes, the forming bundles start out randomly oriented but are then restructured into a largely parallel array (Fig 4D; S7 Movie). Therefore, in addition to SPD-1 bundling microtubules, there is another mechanism working to reorganize these newly formed microtubule bundles into a functional orientation along which chromosomes are able to segregate. One candidate factor that could provide this function is KLP-18, since this motor sorts microtubule bundles during spindle assembly [2], and is present on anaphase spindles following *klp-15/16* depletion (Fig 3C). It is currently unknown whether KLP-18 also functions during anaphase, because the requirement for this protein earlier during spindle assembly has made it difficult to assess an anaphase-specific role; in *klp-18* mutants or RNAi, chromosomes do not segregate into distinct groups in anaphase (Fig 5D) because the spindles are monopolar prior to anaphase onset [4, 12]. However, the KLP-15/16 depletion phenotype offers a unique opportunity to address this question, since this condition has revealed a sorting activity that operates specifically during anaphase to generate parallel arrays of microtubule bundles. Notably, this activity does not require that the microtubules have been sorted previously; following *klp-15/16(RNAi)*, the microtubule bundles start out randomly oriented (Fig 4D) yet they can still be organized into a parallel array. Therefore, this feature allowed us to explore a potential role for KLP-18 during anaphase by co-depleting it with KLP-15/16.

To determine if KLP-18 could be required for this anaphase reorganization activity, we depleted KLP-15/16 in a KLP-18 mutant, *klp-18(tm2841)*, which results in a predicted early stop that is thought to eliminate KLP-18 function [2]. We then stained the spindles for SEP-1 and AIR-2 to stage them as before, to determine if microtubules were able to reorganize into spindles capable of mediating chromosome segregation in late anaphase, as they do following *klp-15/16* depletion in the wild type strain (Figs 3A and 5A). Notably, we found that depletion of KLP-15/16 in the *klp-18(tm2841)* mutant results in a complete failure of microtubule reorganization and chromosome segregation in late anaphase (Fig 5D), despite the fact that SPD-1 was still able to target to the microtubules (Fig 5E) and that the early anaphase configuration appeared similar to KLP-15/16 depletion in the wild-type strain (Figs 3A and S5). These results therefore suggest that KLP-18 could provide the anaphase spindle reorganization activity that we observe in the *klp-15/16(RNAi)* condition. Although we cannot completely rule out the possibility that the metaphase defect in *klp-18* mutant oocytes prevents the microtubule reorganization that normally occurs following KLP-15/16 depletion, we think that our data are at least suggestive that KLP-18 provides this activity during anaphase and that it may therefore have an anaphase role in wild-type spindles. Taken together, we therefore propose that two complementary activities facilitate the reorganization of anaphase spindle microtubules following KLP-15/16 depletion: 1) SPD-1 loads in early anaphase to generate prominent microtubule bundles of mixed polarity, and 2) KLP-18 acts to orient these bundles into a parallel array that is capable of segregating chromosomes.

### Lateral microtubule-chromosome contacts can be established during KLP-15/16-independent anaphase

Finally, we wanted to further investigate the mechanism of chromosome segregation during KLP-15/16-independent anaphase. During wild-type meiosis, microtubule bundles run along the sides of chromosomes prior to anaphase onset. These lateral associations remain in place during anaphase, creating channels that the chromosomes reside in as they move towards spindle poles [4] and then spindle elongation drives chromosomes further apart [7, 8]. Given that microtubules are completely disorganized prior to anaphase onset following KLP-15/16 depletion and, unlike wild-type spindles, have no discernable lateral associations with chromosomes, we wanted to determine what types of microtubule-chromosome contacts were established during anaphase to facilitate segregation.

**Fig 7:**
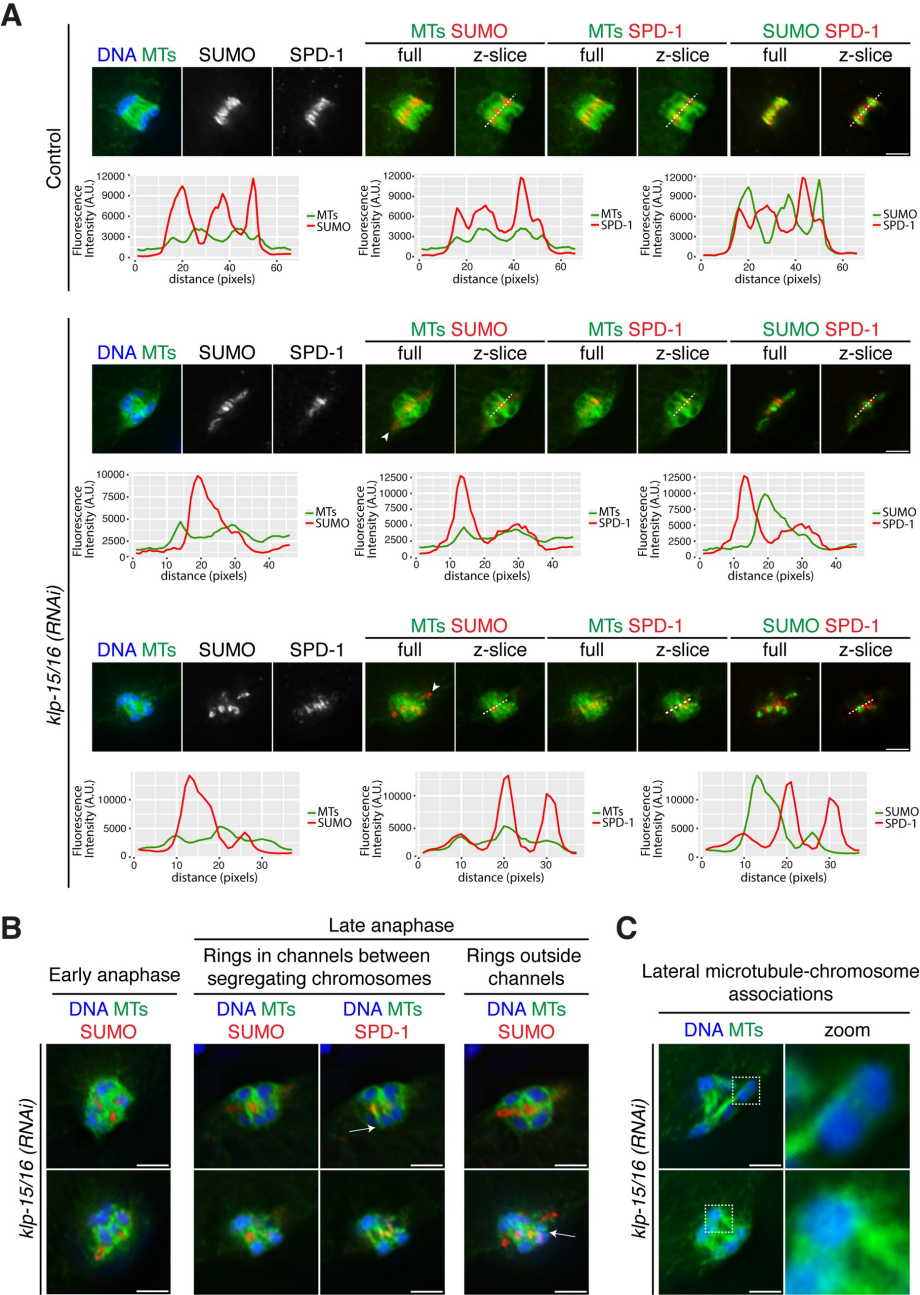
Anaphase spindle organization in *klp-15/16(RNAi)* oocytes. (A) DNA (blue), tubulin (green in columns 1, 4, 5, 6, 7), SUMO (red in columns 4 and 5, green in columns 8 and 9), and SPD-1 (red, columns 6, 7, 8, 9); for each color combination, max projections of the full spindle are labeled “full” and the single z-slices used for the line scans are labeled “z-slice”. In control and *klp-15/16(RNAi)* oocytes, chromosomes can be seen segregating through channels with SUMO in the middle of the channel flanked by SPD-1 on the microtubule bundles. Line scans across the channels in single z-slice images show colocalization of microtubules and SPD-1 and anti-correlation of SUMO/microtubules and SUMO/SPD-1; these oscillations were observed in 9/11 control anaphase spindles (82%) and 12/18 *klp-15/16(RNAi)* anaphase spindles (67%). However, following *klp-15/16(RNAi)*, some rings also appear to be on the periphery of the spindle (arrowheads). (B) Single z-slice images of DNA (blue), tubulin (green), SUMO (red, columns 1, 2, 4), and SPD-1 (red, column 3). In early anaphase following *klp-15/16(RNAi)*, rings are dissociated from chromosomes and are embedded within the microtubules of the spindle, or are excluded from the spindle. In late anaphase, the rings can be within channels between segregating chromosomes or outside of a channel not associated with a segregating chromosome pair. Arrows indicate examples of lateral microtubule-chromosome associations. Within each row, the late anaphase images are different z-slices from the same spindle, chosen to highlight different features. (C) DNA (blue) and tubulin (green); single z-slice images showing additional examples of lateral microtubule-chromosome associations in *klp-15/16(RNAi)* oocytes. We observed clear lateral associations in 31/35 control anaphase spindles (89%) and in 22/31 *klp-15/16(RNAi)* anaphase spindles (71%). Bars = 2.5 *μ*m.

First, we asked if anaphase spindles in *klp-15/16(RNAi)* oocytes are able to form any channels that are analogous to those observed in wild-type oocytes. To this end, we stained spindles for SUMO, to mark the ring structures [36], and SPD-1, to mark anaphase microtubule bundles. In control spindles, each channel is comprised of a pair of separating chromosomes with a ring in between, and SPD-1 marks the microtubule bundles adjacent to the ring. Therefore, line scans of these components in control spindles show an alternating pattern of SUMO/SPD-1 and SUMO/microtubules across the channels (Fig 7A). In *klp-15/16(RNAi)* oocytes, we found similar alternating patterns of these markers in a significant number of spindles (12/18 *klp-15/16(RNAi)* spindles examined; Fig 7A); showing that the spindles are capable of forming microtubule channels during anaphase. Importantly, we also observed microtubules associating laterally with the segregating chromosomes (Fig 7B arrows and Fig 7C; these associations were seen in 22/31 *klp-15/16(RNAi)* spindles) suggesting that this type of association can be established in anaphase, even if these associations are not in place at anaphase onset.

Despite the fact that channels can form during anaphase in *klp-15/16(RNAi)* oocytes, we also found that some rings appeared to be on the periphery of the spindle, demonstrating that not all separating chromosomes end up in a channel with a ring in the center (Fig 7A, arrowheads). To gain insight into this variability, we looked earlier in anaphase before the microtubules were reorganized into bundles. In early anaphase spindles, at the “microtubule ball” stage when homologous chromosomes first begin to come apart, we observed some rings embedded in the microtubule ball close to the separating chromosomes, but also some rings towards the periphery of the structure (Fig 7B). Subsequently, when the microtubules are bundled and aligned into parallel arrays, rings can be seen both in channels between segregating chromosomes and also completely outside of the reorganized spindle (Fig 7B). This is likely due to the fact that microtubule bundling and reorganization are occurring as chromosomes begin to come apart. This results in some chromosomes and rings becoming organized within channels while others are not. This behavior may also contribute to the presence of lagging chromosomes in these spindles (Figs 3B, 5A and 7A). Therefore, while it is unlikely that the complete formation of a ring-containing channel is essential for chromosome segregation, the fact that lateral associations are established suggests that they could contribute to segregation in this context.

## Discussion

### Minus-end kinesins stabilize microtubule bundles in *C*. *elegans* acentriolar meiotic spindles

**Fig 8:**
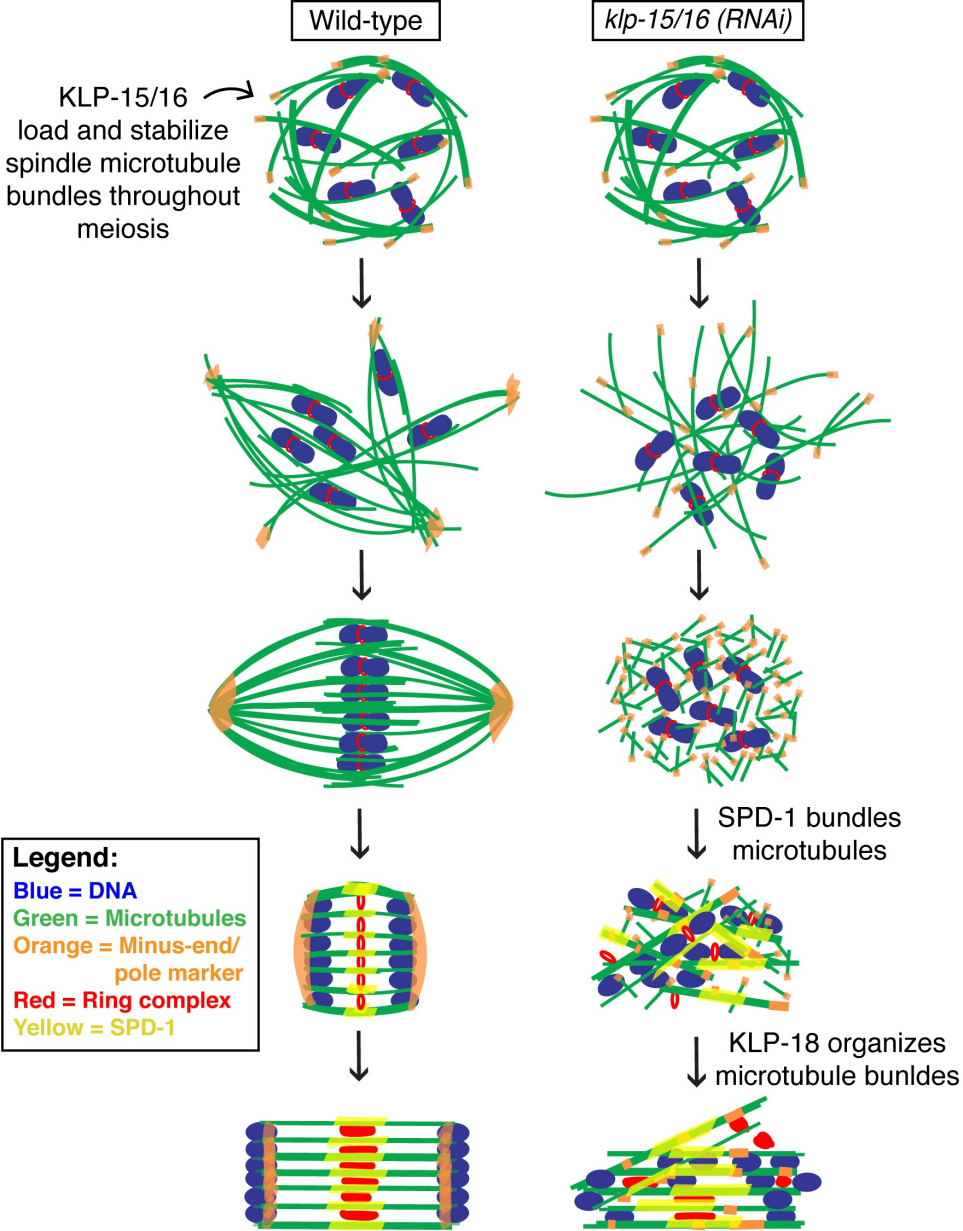
Interplay between microtubule-associated factors contributes to acentriolar spindle assembly and maintenance. DNA (blue), microtubules (green), minus-end/pole marker (orange), ring complex (red), and SPD-1 (yellow). In wild-type spindles, KLP-15/16 load onto microtubules during the cage stage and remain associated through anaphase providing stability to microtubule bundles. At anaphase onset, SPD-1 loads onto the microtubules as the chromosomes begin to segregate towards the poles through microtubule channels (Anaphase A), and the ring complexes are left behind inside the channels. As anaphase progresses, the spindle microtubules elongate forcing the chromosomes further apart (Anaphase B), and the ring complexes disassemble. In *klp-15/16(RNAi)* oocytes, after the cage stage, microtubules form a disorganized array with ASPM-1 located throughout the structure. The array collapses into a microtubule ball around the chromosomes with ASPM-1 throughout. As anaphase begins, SPD-1 binds to the short, randomly oriented microtubules and bundles them, the ring complexes dissociate from the chromosomes, and KLP-18 sorts and aligns the bundles such that lateral microtubule-chromosome associations and channels can form. The spindle then elongates (Anaphase B) to facilitate chromosome segregation.

Taken together, our data have revealed two distinct mechanisms that act to bundle microtubules in acentriolar spindles. Prior to spindle assembly, KLP-15/16 localize diffusely throughout the cytoplasm, which is in contrast to kinesin-14s from other organisms that have been shown to be sequestered in the nucleus [37, 38]. Then, as acentriolar spindles begin to form, KLP-15/16 load onto microtubule bundles during the cage stage, stabilizing them to facilitate spindle assembly (Fig 8). This proposed function is consistent with previous studies of kinesin-14s in other organisms, which demonstrated that this family of kinesins is required for acentriolar spindle formation and localize uniformly to acentriolar spindle microtubules [30, 39–41]. However, while depletion of kinesin-14s in other organisms predominantly results in defects such as unfocused poles and splayed microtubules, depletion of KLP-15/16 in *C*. *elegans* oocytes completely prevents bipolar spindle formation and abolishes microtubule bundling prior to anaphase, implicating these proteins in the stabilization of microtubule bundles comprising the acentriolar meiotic spindle. Furthermore, unlike most other organisms where kinesin-14s perform essential roles in mitosis [40, 42–44], KLP-15/16 are not required for mitotic spindle formation in *C*. *elegans*, suggesting a unique role for KLP-15/16 that is specific to acentriolar spindle assembly; this finding is also reminiscent of studies in *Drosophila*, where inhibition of the kinesin-14 Ncd has a much more severe phenotype in oocytes than it does in mitosis [38, 42, 45]. The presence of the “microtubule ball” comprised of short microtubules that ultimately forms prior to anaphase following *klp-15/16(RNAi)* raises the intriguing possibility that the function of KLP-15/16 could be to stitch together short microtubules into longer bundles that can then be sorted and organized into a bipolar spindle. Since kinesin-14s contain a motor domain in the C-terminus and a microtubule binding domain in the N-terminus [37, 43, 46], and it has been reported that this class of kinesins can stabilize and cross-link microtubules in a parallel configuration [47], it is possible that these motors could contribute to this stitching activity. This interpretation is consistent with a previous electron microscopy study, which reported the presence of many short microtubules in a partial reconstruction of a *C*. *elegans* oocyte spindle [48], and also with a study in *Xenopus* egg extracts that demonstrated that meiotic spindles are comprised of tiled arrays of short microtubules [49]; therefore a microtubule stitching activity might be something that is especially important in the context of acentriolar meiosis. Our studies suggest that KLP-15/16 could be factors that organize these short microtubules into longer bundles capable of mediating chromosome congression and segregation.

### New insights into chromosome segregation in acentriolar meiosis

In addition to revealing an important function for KLP-15/16, our studies have yielded insights into previously unknown mechanisms promoting accurate chromosome segregation during acentriolar meiosis. First, we found that the PRC1-family protein SPD-1 provides a secondary activity that stabilizes microtubule bundles during anaphase (Fig 5A, 5B and 5C). This activity was previously unidentified, as prior depletion of SPD-1 [8], confirmed by our own studies (Fig 5A), failed to reveal an obvious anaphase defect. However, this is likely because KLP-15/16 are present on the anaphase spindle stabilizing the microtubule bundles (Fig 2B), making SPD-1 non-essential until these proteins are depleted. This discovery is similar to findings in fission yeast, where the SPD-1 homolog Ase1 is not essential on its own but provides a backup mechanism for bipolar spindle assembly under conditions where the kinesin-5 motor Cut7 and the kinesin-14 motor Pkl1 are deleted [50].

During mitosis in other organisms, homologs of SPD-1 are known to crosslink antiparallel microtubules [51–53], and our data are consistent with SPD-1 performing a similar function in *C*. *elegans* oocytes. During wild-type meiotic anaphase, this protein loads onto the central region of the spindle (Fig 4D), where this putative crosslinking activity could reinforce anaphase spindle structure. Under KLP-15/16 depletion conditions, SPD-1 loads at the microtubule ball stage (Fig 4D), which contains many short microtubules that are likely randomly oriented (Fig 1C); given this configuration, the ability to crosslink antiparallel microtubules would enable SPD-1 to bundle microtubules. These SPD-1-stabilized microtubule bundles could then be sorted and aligned into a parallel array by the action of KLP-18. Therefore, our studies have uncovered a new function for SPD-1 on *C*. *elegans* acentriolar spindles, and also represent the first demonstration that PRC1-family proteins play a role during oocyte meiosis. Furthermore, our work also suggests that KLP-18 may be functional during anaphase in these cells, since it appears to organize the microtubule bundles generated by SPD-1 (Fig 5D and 5E), suggesting that this motor not only plays roles during bipolar spindle formation, but may also be required to maintain spindle organization as chromosomes segregate.

Finally, our work has also shed light on the mechanisms driving chromosome segregation in this unique form of anaphase. We found that anaphase spindles in KLP-15/16-depleted oocytes are sometimes able to form channels with lateral microtubule-chromosome associations (Fig 7), despite the lack of microtubule bundles prior to anaphase onset. These data suggest that this form of microtubule-chromosome contact is preferred during anaphase and points to a role for the chromosomes providing significant structural cues for spindle organization as these associations can be established *de novo* following KLP-15/16 depletion. However, given that these laterally-associated bundles may be comprised of microtubules of mixed polarity (Fig 3B and 3C), it is improbable that they would be able to efficiently facilitate directional chromosome movement (a mechanism we proposed to drive normal Anaphase A [4]). Therefore, we suggest that the primary force driving segregation in the absence of KLP-15/16 is the elongation of these lateral bundles in an Anaphase-B type mechanism.

The discovery that lateral associations are established during anaphase is also interesting since two other types of chromosome-spindle contacts have been proposed to facilitate segregation during wild-type anaphase: 1) elongating microtubules contacting the inside surfaces of separating chromosomes to provide a pushing force [8] and 2) chromosomes contacting the spindle poles, so that outward pole separation can drive segregation in Anaphase B [7]. It is possible that the first type of association contributes to segregation during KLP-15/16-independent anaphase; since not every chromosome ends up in a normal microtubule channel (Fig 7), some microtubules might randomly make contacts with chromosome surfaces and provide a pushing force, contributing to segregation alongside the bundles that are laterally-associated. Indeed, our data are consistent with this idea since we observe non-laterally-associated microtubules in the reorganized *klp-15/16(RNAi)* anaphase spindles (Fig 7). In contrast, the second model proposed that spindle shrinkage enables the chromosomes to establish a physical tether to a cross-linked network of microtubules and pole proteins; outward sliding of interpolar microtubules would then drive the poles and the tethered chromosomes apart [7]. Our observation that pole proteins KLP-18 and ASPM-1 are distributed throughout *klp-15/16(RNAi)* spindles both prior to and during anaphase (Figs 1C, 1D, 3B and 3C) makes it difficult to imagine how such a tether would efficiently form in this context, and we therefore speculate that pre-established spindle poles may not be absolutely required to segregate chromosomes in *C*. *elegans* oocytes (although we cannot rule out the possibility that other pole proteins exhibit a higher level of organization in these mutant spindles). Moreover, our data also raise the possibility that spindle elongation could be capable of driving segregation even when the polarity of microtubules within the spindle is disrupted, potentially revealing an unusual mode of chromosome segregation that operates in this mutant context.

In summary, our studies have uncovered a crucial role for KLP-15 and KLP-16 in *C*. *elegans* acentriolar spindle assembly, revealed a second, anaphase-specific mechanism dependent on SPD-1 operating in parallel to these kinesins, and provided new insights into anaphase spindle organization and chromosome segregation mechanisms during acentriolar meiosis.

## Materials and methods

### Strains

In this study, ‘wild-type’ refers to N2 (Bristol) or EU1067 worms grown on NGM/OP50 plates, and ‘control’ refers to the RNAi vector control (L4440).

### Strain list

N2 (Bristol)

ANA065: *adeIs1[pMD191*, *mex-5*::*spd-1*::*GFP]* II (gift from Marie Delattre)

ANA072: *adeIs1[pMD191*, *mex-5*::*spd-1*::*GFP]* II; *ltIs37[pAA64; pie-1*::*mCherry*::*his-58; unc-119(+)]* IV (gift from Marie Delattre)

EU716: *zen-4(or153)* IV (from the CGC). For experiments using *zen-4(or153)*, plates were shifted to 25°C 16–18 hours before dissection and fixation.

EU1067: *unc-119(ed3) ruIs32[unc-119(+) pie-1*::*GFP*::*H2B] III; ruIs57[unc-119(+) pie-1*::*GFP*::*tubulin]* (gift from Bruce Bowerman)

OD57: *unc-119(ed3) III; ltIs37[pAA64; pie-1*::*mCherry*::*his-58; unc-119(+)]* IV; *ltIs25[pAZ132; pie-1*::*GFP*::*tba-2; unc-119 (+)]* IV (gift from Arshad Desai)

RB1593: *klp-15(ok1958)* I. *ok1958* is a deletion allele of the last 391 amino acids of KLP-15 (from the CGC)

SMW13: *klp-18(tm2841)IV/nT1[qIs51*]; *unc-119(ed3) ruIs32[unc-119(+) pie-1*::*GFP*::*H2B] III; ruIs57[unc-119(+) pie-1*::*GFP*::*tubulin]* (Wolff et. al., 2016)

SMW15: *klp-16(wig1)* I. This strain was generated using a CRISPR approach detailed below.

SMW16: *Pklp-16*::*klp-16*::*GFP (C1971>A*–*PAM site mutation)* I. This strain was generated using a CRISPR approach detailed below.

SMW18: (SMW16 x OD56) *Pklp-16*::*klp-16*::*GFP (C1971>A–PAM site mutation) I; ltIs37 [(pAA64) pie-1*::*mCherry*::*his-58 + unc-119(+)] IV*

### Generation of KLP-16::GFP strain

A CRISPR-based approach [54, 55] was used to generate an endogenously tagged KLP-16::GFP strain (SMW16). Briefly, 27μM recombinant Cas9 protein (IDT) was co-injected with 13.6μM tracrRNA (IDT), 4μM *dpy-10* crRNA (5’—GCUACCAUAGGCACCACGAG- 3’) (IDT), 1.34μM *dpy-10* repair oligo (Ultramer from IDT; 5’ -CACTTGAACTTCAATACGGCAAGATGAGAATGACTGGAAACCGTACCGCATGCGGTGCCTATGGTAGCGGAGCTTCACATGGCTTCAGACCAACAGCCTAT- 3’), 9.6μM *klp-16* crRNA (5’—UGUCUAGUUCAUAGACAUCU- 3’) (IDT); and 136ng/μL ssDNA *klp-16* repair template into N2 worms, that were then allowed to produce progeny. Worms from plates containing rollers and dumpys were screened for GFP expression, and homozygous KLP-16::GFP worms were identified by PCR screening. To make the *klp-16* repair template, we generated a C-terminal LAP tag using a GBlock (IDT) and Gibson Assembly to create an S-TEV-GFP construct. The tag was then amplified using PCR with primers that contained homology to the *klp-16* gene with the final product containing 68 bp of homology upstream of the *klp-16* stop codon and 100 bp of homology downstream of the stop codon. ssDNA was generated by asymmetric PCR. SMW16 (KLP-16::GFP) was also crossed with OD56 (mCherry::histone) to generate SMW18: *Pklp-16*::*klp-16*::*GFP (C1971>A—PAM site mutation) I; ltIs37 [(pAA64) pie-1*::*mCherry*::*his-58 + unc-119(+)] IV*.

### Generation of *klp-16(wig1)* strain (SMW15)

A CRISPR-based approach similar to the one above was used to generate a worm strain with a ~600 bp deletion in the *klp-16* locus beginning ~100 bp upstream of the start codon. Essentially the same approach was used as above; the differences being two crRNAs (4.8μM) (5’- AGGCGGAGUUUAAGUUUGAG-3’ and 5’- CUCCUCAAGAAGCGUCACUU-3’) (IDT) (one upstream and one downstream of the *klp-16* start codon, respectively), and a ssDNA oligo (4μM) (Ultramer from IDT; 5’-CAGCCATCTCACGCTCCAATTGCGCATTTCTCTCCTCAAGAAGCGTCACTTCTCAAACTTAAACTCCGCCTCTGAAAATTCCCGCCAAATCGGATGGATTAC-3’) were used in the injection mix. The repair Ultramer sequence is homologous to the sequence just upstream and downstream to the two CRISPR cut sites thereby deleting the ~600 base pairs. Worms from plates containing rollers and dumpys were screened by PCR and homozygous mutants were isolated.

### KLP-15/16 domain analysis

Protein domains were determined using PsiPred [56] and Paircoil2 [57]. Protein sequences were analyzed using Clustal Omega [58]. Proline-rich regions of proteins have been shown to bind microtubules [46]. The proline content of amino acids 1–149 is 14% for KLP-15 and 13% for KLP-16.

### RNAi

From a feeding library [26, 59], individual RNAi clones were picked and grown overnight at 37°C in LB with 100μg/ml ampicillin. Overnight cultures were spun down and plated on NGM (nematode growth media) plates containing 100μg/ml ampicillin and 1mM IPTG. Plates were dried overnight. Worm strains were synchronized by bleaching gravid adults and letting the eggs hatch overnight without food. L1s were then plated on RNAi plates and grown to adulthood at 15° for 5–6 days.

### Embryonic lethality

Young adult worms grown on control plates or plates containing RNAi-expressing bacteria were transferred to new plates containing either control or RNAi-expressing bacteria and allowed to lay eggs for 24 hours at 15°C before being moved to another fresh plate of either control or RNAi-expressing bacteria. The eggs were allowed to hatch for 24 hours and then the progeny (eggs and hatched worms) were counted. For each parent worm this process was repeated twice, resulting in three days of progeny being counted. For each condition, the progeny of at least 15 worms were scored.

### Immunofluorescence and antibodies

Immunofluorescence was performed by freeze cracking embryos and plunging into -20°C methanol as described [60]. Embryos were fixed for 35–45 minutes, rehydrated in PBS, and blocked in AbDil (PBS plus 4% BSA, 0.1% Triton X-100, 0.02% Na-Azide) for 30 minutes. Primary antibodies were incubated overnight at 4°C. The next day, embryos were washed 3x with PBST (PBS plus 0.1% Triton X-100), incubated in secondary antibody for 1 hour and 15 minutes, washed again as before, incubated in mouse anti-α-tubulin-FITC for 1.5 hours, washed again, and incubated in Hoechst (1:1000 in PBST) for 15 minutes. Embryos were then washed 2x with PBST, mounted in 0.5% *p*-phenylenediamine, 20mM Tris-Cl, pH 8.8, 90% glycerol or ProLong Gold antifade Mountant (Molecular Probes), and sealed with nail polish; except for the overnight primary, the entire procedure was performed at room temperature. For experiments using the rabbit anti-KLP-16 antibody and staining of SPD-1::GFP with mouse anti-GFP, embryos were blocked in AbDil overnight at 4°C and incubated in primary antibody for 2 hours at room temperature. Primary antibodies used in this study: rabbit anti-ASPM-1 (1:5000, gift from Arshad Desai), rabbit anti-SEP-1 (1:400; gift from Andy Golden), rabbit anti-KLP-18 (1:10,000, gift from O. Bossinger), rabbit anti-ZEN-4 (1:500; gift from Michael Glotzer), mouse anti-SUMO (1:500; gift from Federico Pelisch), mouse anti-GFP (1:200; Invitrogen). Rat anti-AIR-2 was generated by Covance using the C-terminal peptide sequence KIRAEKQQKIEKEASLRNH (synthesized by the Peptide Synthesis Core Facility at Northwestern University), then affinity purified and used at 1:1000. Rabbit anti-KLP-16 was generated by Covance using the N-terminal peptide sequence CMNVARRRSGLFRSTIGAPPK (synthesized by the Peptide Synthesis Core Facility at Northwestern University), then affinity purified and used at 1:2000. Rabbit anti-SPD-1 was generated by Proteintech using the C-terminal peptide sequence CIASSTPSSAKKVLTRRNQFL, then affinity purified and used at 1:1000. Directly conjugated mouse anti-α-tubulin-FITC (DM1α, Sigma) and Alexa-fluor directly conjugated secondary antibodies (Invitrogen) were used at 1:500. All antibodies were diluted in AbDil.

### Microscopy

All fixed imaging and high resolution imaging of KLP-16::GFP and KLP-16::GFP;mCherry::histone was performed on a DeltaVision Core deconvolution microscope with a 100x objective (NA = 1.4) (Applied Precision). This microscope is housed in the Northwestern University Biological Imaging Facility supported by the NU Office for Research. Image stacks were obtained at 0.2μm z-steps and deconvolved using SoftWoRx (Applied Precision). All images in this study were deconvolved and displayed as full maximum intensity projections of data stacks encompassing the entire spindle structure, unless stated otherwise. For KLP-16::GFP and KLP-16::GFP;mCherry::histone imaging, live worms were mounted in anesthetic (0.2% tricaine, 0.02% levamisole in M9).

### Time-lapse imaging

Two-color live imaging was performed using a spinning disk confocal microscope with a 63x HC PL APO 1.40 NA objective lens. A spinning disk confocal unit (CSU-X1; Yokogawa Electric Corporation) attached to an inverted microscope (Leica DMI6000 SD) and a Spectral Applied Imaging laser merge ILE3030 and a back-thinned electron-multiplying charge-coupled device (EMCCD) camera (Photometrics Evolve 521 Delta) were used for image acquisition. The microscope and attached devices were controlled using Metamorph Image Series Environment software (Molecular Devices). Typically, ten to twelve z-stacks at 1μm increments were taken every 20–45 seconds at room temperature. Image deconvolution was done using AutoQuant X3 (Media Cybernetics Inc.). Images were processed using ImageJ. Images are shown as maximum intensity projections of entire spindle structure. Live, intact worms were mounted on 5% agarose, M9 pads in 50% live imaging solution (modified S-basal [50mM KH2PO4,10mM K-citrate, 0.1M NaCl, 0.025mg/ml cholesterol, 3mM MgSO4, 3mM CaCl2, 40mM serotonin creatinine sulfate monohydrate]), 50% 0.1 micron polystyrene Microspheres (Polysciences Inc.), and covered with a coverslip. The spinning disk microscope is housed in the Northwestern University Biological Imaging Facility supported by the NU Office for Research.

For S3 Movie, EU1067 worms were picked into a solution of tricaine (2%) and tetramisole (0.4%), and incubated for ~30 min. Worms were then pipetted onto a 3% agarose pad, covered with a coverslip, and imaged immediately on a DeltaVision Core deconvolution microscope (same as above). Image stacks were obtained at 1μm z-steps at 10 second intervals using 2 × 2 binning, and then deconvolved. Video images are full projections of data stacks.

### Image analysis and quantification

**Fig 1D:** Slides made on the same day were imaged within an 8 hour window on a DeltaVision Core deconvolution microscope (see Microscopy section) using the same exposure conditions and times for all slides. In ImageJ, linescans of 154 x 75 pixels (L x W) were performed on 6 z-slice sum projections of representative spindles from control (n = 8) and *klp-15/16(RNAi)* (n = 9) embryos. In control spindles, the linescans were done along the pole-to-pole axis. In spindles from *klp-15/16(RNAi)* embryos, linescans were done straight along the x-axis of the image, since these spindles lack a discernable orientation. The average fluorescence intensity for each channel was graphed (solid line) along with the SEM (standard error of the mean) (shaded area) using the ggplot package in R Studio. The y-axes of the graphs are the same between control and experiment for a given channel.

**Fig 2B:** α-KLP-16 staining for each stage of spindle assembly in wild-type oocytes/embryos. Oocytes in prophase with the nuclear envelope intact were scored as “localized” if the KLP-15/16 signal was primarily cytoplasmic. During all other stages, oocytes were scored as “localized” if the KLP-15/16 signal was colocalized with spindle microtubules. The quantification is as follows: diakinesis 81.8% (n = 22), cage 0% (n = 13), multipolar 31.3% (n = 67), bipolar 64% (n = 114), anaphase 51.9% (n = 79), mitotic spindles 3.7% (n = 27). Although not every spindle is stained, we think that this represents variability with the immunofluorescence procedure and with the antibody (since we see 100% localization of KLP-16::GFP to oocyte spindle microtubules and to mitotic spindles; see S4A Fig).

**Fig 2C:** α-KLP-16 staining was scored in *klp-15/16(RNAi)* oocytes. The number of oocytes in which we could discern any spindle staining is as follows: microtubule ball stage 8% (n = 75), anaphase 2.9% (n = 35). α-KLP-16 staining was scored in *klp-15(ok1958)* oocytes. Oocytes in prophase with the nuclear envelope intact were scored as “localized” if the KLP-15/16 signal was primarily cytoplasmic. During all other stages, oocytes were scored as “localized” if the KLP-15/16 signal was colocalized with spindle microtubules. The quantification is as follows: diakinesis 100% (n = 3), cage 14% (n = 7), multipolar 44% (n = 25), bipolar 72.2% (n = 18), anaphase 38% (n = 21). α-KLP-16 staining was scored in *klp-16(wig1)* oocytes as above. The quantification is as follows: diakinesis 100% (n = 10), cage 42.9% (n = 7), multipolar 89% (n = 19), bipolar 81.8% (n = 11), anaphase 79.2% (n = 24). As with the control strain (see Fig 2B quantification above), we think that the incomplete staining we observe is due to variability with the procedure and antibody.

**Fig 3B:** Linescans of control anaphase spindles and anaphase spindles from *klp-15/16(RNAi)* oocytes stained for ASPM-1 were performed using the arbitrary profile tool in SoftWorx (Applied Precision). A spindle was scored as having staining at poles if the ASPM-1 signal was enriched at two ends of the spindle near the segregating chromosomes. ASPM-1 was enriched at the poles of 21/24 control spindles, but was largely diffuse along spindle microtubules in anaphase of *klp-15/16(RNAi)* oocytes (only 9/26 spindles could be classified as having any type of ASPM-1 enrichment, and this enrichment was not as strong as in the control spindles).

**Fig 3C:** Linescans of control anaphase spindles and anaphase spindles from *klp-15/16(RNAi)* oocytes stained for KLP-18 were performed using the arbitrary profile tool in SoftWorx (Applied Precision). A spindle was scored as having staining at poles if the KLP-18 signal was enriched at two ends of the spindle near the segregating chromosomes. KLP-18 was enriched at poles in 11/12 control spindles but was diffuse in *klp-15/16(RNAi)* spindles (0/6 had KLP-18 concentrated into poles).

**Fig 3D:** Aneuploidy in MII embryos was quantified by counting the number of chromosomes in MII in immunofluorescence images of control and *klp-15/16(RNAi)* embryos. An embryo was scored as ‘aneuploid’ if the number of chromosomes was not 6.

**Fig 4A and 4B:** ZEN-4 and SPD-1 staining was scored in control spindles and spindles from *klp-15/16(RNAi)* oocytes. Staining of metaphase and anaphase spindles (staged by AIR-2 localization) was scored for each condition. ZEN-4: Control metaphase 1.6% (n = 63), Control anaphase 90% (n = 20); *klp-15/16(RNAi)* metaphase 2.9% (n = 134), *klp-15/16(RNAi)* anaphase 80.3% (n = 66). SPD-1: Control metaphase 9.5% (n = 42), Control anaphase 97% (n = 66); *klp-15/16(RNAi)* metaphase 1% (n = 100), *klp-15/16(RNAi)* anaphase 88.2% (n = 85).

**Fig 5A and 5B:** Quantification of microtubule bundling and chromosome segregation was done using immunofluorescence images of anaphase spindles with SEP-1 gone and AIR-2 relocalized to the microtubules (mid/late anaphase) for the conditions shown. We scored microtubule bundling by eye, looking through the entire z-stack in SoftWorx (Applied Precision). An anaphase spindle was scored as “bundled” if one or more microtubule bundles were discernable. Chromosomes were scored as “segregated” if two or more distinct masses of chromosomes were observed. The simple matching coefficient (SMC) for microtubule bundling and chromosome segregation = 0.82 (n = 251); in other words, 82% of the spindles were scored as microtubules bundled and chromosomes segregated or as no microtubule bundles and no chromosome segregation.

**Fig 5C:** To approximate anaphase microtubule lengths, we used the measure distances tool in SoftWorx (Applied Precision). Using this tool, a line was manually drawn (point by point) along the most prominent spindle microtubule bundle through the 3D stack of an image to measure its full length.

**Fig 6A and 6B:** Polar body number and maternal pronuclei number were quantified by scoring live EU1067 worms mounted in anesthetic (0.2% tricaine, 0.02% levamisole in M9) on a Leica DM5500B fluorescent microscope.

**Fig 7A:** Linescans of control anaphase spindles and anaphase spindles from *klp-15/16(RNAi)* oocytes stained for tubulin, SUMO, and SPD-1 were performed using the arbitrary profile tool in SoftWorx (Applied Precision). A spindle was scored as having oscillations if one or more instances of alternating MTs/SUMO and SUMO/SPD-1 signal was observed. This analysis was done by examining both single z-slices and max projections of spindles. Oscillations were observed in 9/11 control anaphase spindles and 12/18 *klp-15/16(RNAi)* anaphase spindles.

**Fig 7C:** Lateral microtubule associations to chromosomes were scored in control anaphase spindles and anaphase spindles from *klp-15/16(RNAi)* oocytes. A spindle was scored as having lateral microtubule/chromosome associations if a microtubule appeared to contact and run along the side of a chromosome. This analysis was done by examining both single z-slices and max projections of spindles. We observed clear lateral associations in 31/35 control anaphase spindles and in 22/31 *klp-15/16(RNAi)* anaphase spindles.

**S2C Fig:** Live, intact worms expressing GFP::tubulin, GFP::histone (EU1067) fed either control or *klp-16(RNAi)*-expressing bacteria were anesthetized in 0.2% tricaine, 0.02% levamisole in M9 and viewed on a Leica DM5500B widefield fluorescence microscope. Spindles in embryos in the -1, spermatheca, and +1 positions within the gonad were scored for microtubule organization by eye. A spindle was scored as “multipolar” if it had prominent microtubule bundles that formed more than two organized poles. A spindle was scored as an “array” if the microtubule structure lacked prominent bundles and organized poles. A spindle was scored as “MT ball” if the microtubule structure had collapsed around the chromosomes and lacked prominent bundles and organized poles.

**S3B Fig:** Spindle volumes were measured using the surfaces tool in Imaris (Bitplane). Using the full 3D image stack, this tool renders a 3D surface based on fluorescence signal (for our analysis, we used the tubulin signal). The volume of this 3D surface is then measured. The volumes of metaphase and early anaphase spindles (staged by SEP-1/AIR-2 localization) from *klp-15/16(RNAi)* oocytes were measured and compared to the volumes of the spindles used for the linescan measurements in Fig 1D. This analysis allowed us to conclude that the spindles used in our linescans for Fig 1D are within the range of metaphase spindles based on spindle volume.

**S4A Fig:** Live, intact worms expressing KLP-16::GFP, mCherry::histone (SMW18) were anesthetized in 0.2% tricaine, 0.02% levamisole in M9 and viewed on a Leica DM5500B widefield fluorescence microscope. The localization of KLP-16::GFP was scored in oocytes/embryos in the -1, spermatheca, and +1 positions within the gonad. KLP-16::GFP signal was scored as cytoplasmic if it was absent/reduced in the nucleus. Because the localization of KLP-16::GFP on the spindle looks identical to the organization of GFP::tubulin, we scored localization for the following categories: cage, multipolar, bipolar, and anaphase. The organization of the chromosomes visualized by mCherry::histone was used to identify and better stage the spindles.

**S4C Fig:** α-GFP staining for each stage of spindle assembly in SMW16 (KLP-16::GFP). Oocytes in prophase with the nuclear envelope intact were scored as “localized” if the α-GFP signal was primarily cytoplasmic. During all other stages, oocytes were scored as “localized” if the α-GFP signal was colocalized with spindle microtubules. The quantification is as follows: diakinesis 100% (n = 2), cage 0% (n = 4), multipolar 76.5% (n = 17), bipolar 96.2% (n = 26), anaphase 83.3% (n = 24), mitotic spindles 100% (n = 5). As with the KLP-15/16 antibody, we think that the lack of staining in all embryos represents variability with the immunofluorescence procedure, since we see robust localization of KLP-16::GFP to microtubules when we visualize this strain live.

### Western blotting

Seventy-five EU1067, RB1593, or SMW15 worms were picked off of control, *klp-16(RNAi)* (EU1067 and SMW15), or *klp-15(RNAi)* (RB1593) plates onto new, empty (no bacteria) plates. The worms were washed off the plates with cold M9 and transferred to a 1.5ml microcentrifuge tube. Worms were pelleted by spinning at 2000 rpm for 1 minute, and the tube was put on ice for ~2 minutes to allow worms to slow down and form a tight pellet. The M9 was removed and the tube was filled with fresh, cold M9 and mixed. The worms were washed a total of 3 times. After the final wash, as much M9 was removed as possible and 2X SDS sample buffer was added to the remaining worm/M9 mixture and boiled for 10 minutes. Samples were run on a 10% SDS-PAGE gel and blotted. For western analysis, rabbit anti-KLP-16 antibody (1:10,000) and mouse anti-tubulin (1:5000) (Sigma, DM1α) were used.

## Supporting information

S1 Fig Amino acid sequence alignment of KLP-15 and KLP-16. Residues shaded in green are identical between the two proteins. Prolines in the proline-rich tail (1–149 aa) are denoted with asterisks. The dotted line denotes the region of complementarity to *klp-15(RNAi)* and the dashed line denotes the region of complementarity to *klp-16(RNAi)*. The solid line under the N-terminal 20 amino acids marks the peptide sequence that was used to make our KLP-15/16 antibody; the red shaded residue is the single amino acid difference between KLP-15 and KLP-16 in the sequence used to make the antibody.
(TIF)

S2 Fig Additional quantification of phenotypes in mutants and following *klp-15/16(RNAi)*. (A) Results of embryonic lethality assays from wild-type, *klp-15(ok1958)*, *and klp-16(wig1)* worms. The wild-type and *klp-16(wig1)* worms were fed bacteria expressing the RNAi clone annotated as targeting KLP-16, and *klp-15(ok1958)* worms were fed bacteria expressing the RNAi clone annotated as targeting KLP-15. (B) Diagram of the *C*. *elegans* germline. The germline is organized in an assembly-line fashion where oocytes in prophase (-3 to -1 positions) are ovulated into the spermatheca where they are fertilized. These fertilized embryos begin the meiotic divisions and exit the spermatheca to the +1 position where they continue to progress through meiosis and subsequently mitosis. This organization enables staging of spindles based on the position of the oocyte/embryo in the germline. (C) Quantification of spindle phenotypes in control and *klp-15/16(RNAi)* worms. This analysis was done using live worms expressing GFP::tubulin, GFP::histone. n represents the number of oocytes/embryos analyzed for each condition.
(TIF)

S3 Fig Additional analysis of spindle defects in mutants and following *klp-15/16(RNAi)*. (A) Shown are DNA (blue), microtubules (green), and ASPM-1 (red) for the nine *klp-15/16(RNAi)* microtubule ball images used for the linescan analysis performed in Fig 1D. ASPM-1 sometimes displays areas of concentration within these structures (examples denoted with arrowheads), that could get averaged out in the graph shown in Fig 1D due to the heterogeneity of the structures. However, we did not observe any clear examples where microtubules appeared to be well-organized into ASPM-1-rich poles that resembled those in wild type spindles, demonstrating that spindle organization is disrupted. (B) Boxplot of spindle volumes of metaphase, early anaphase and the spindles that were used for the linescan analysis in Fig 1D. Metaphase and early anaphase spindles were staged by SEP-1 and AIR-2 localization. Shaded bars represent the range of volumes, bar within the boxes represents the mean, and n represents the number of spindles analyzed for each condition. The range of volumes of the spindles used for linescans suggest that our analysis included both metaphase and early anaphase spindles. (C) DNA (blue), microtubules (green), ASPM-1 (red). In both *klp-15(ok1598)* and *klp-16(wig1)* oocytes, spindles are indistinguishable from wild-type spindles. n represents the number of spindles observed for each condition. Bars = 2.5 *μ*m.
(TIF)

S4 Fig Additional analysis of KLP-16::GFP localization. (A) Quantification of KLP-16 localization in live worms expressing KLP-16::GFP and mCherry::histone (SMW18). KLP-16 localization was scored as “cytoplasmic” if the GFP signal was absent from inside the nucleus. At all other stages, the GFP signal was enriched on the spindle near the chromosomes. n represents the number of oocytes/embryos analyzed for each condition. (B and C) Examples of KLP-16 localization in live worms expressing KLP-16::GFP (SMW16). (B) KLP-16::GFP is absent from the nucleus prior to spindle assembly. During spindle assembly, KLP-16 localizes to the spindle microtubule bundles and remains associated through anaphase; the image of the cage stage is a sum projection of the spindle structure. (C) KLP-16 localizes to mitotic spindle microtubules and centrosomes in one-cell stage embryos. (D and E) DNA (blue), microtubules (green), GFP (red). Meiotic (D) and mitotic (E) spindles from worms expressing KLP-16::GFP (SMW16) were stained with an anti-GFP antibody. The staining pattern of KLP-16::GFP on meiotic spindles (D) is identical to the staining pattern using the anti-KLP-15/16 antibody. (E) KLP-16::GFP was detected on spindle microtubules and centrosomes (quantification in Materials and Methods). The mitotic image was not deconvolved. Bars = (B and D) 2.5 μm; (C and E) 10 μm.
(TIF)

S5 Fig Early anaphase in *klp-18(tm2841)*. DNA (blue), tubulin (green), AIR-2 (not in merge), and SEP-1 (red in merge). In the *klp-18(tm2841)* mutant strain, early anaphase spindles (where SEP-1 is colocalized with AIR-2) begin as a ball of microtubules surrounding the chromosomes, in both control and *klp-15/16(RNAi)* treated oocytes. n represents the number of spindles observed for each condition.
(TIF)

S1 Movie S1 Movie shows spindle assembly in control (left) and *klp-15/16(RNAi)* (right) oocytes expressing mCherry::histone; GFP::tubulin and corresponds to Fig 1B. Bar = 10 μm.
(MOV)

S2 Movie S2 Movie shows another example of spindle assembly in a *klp-15/16(RNAi)* embryo expressing mCherry::histone; GFP::tubulin. Bar = 10 μm.
(MOV)

S3 Movie S3 Movie shows a movie stepping through and rotating around a control spindle stained for DNA (blue), microtubules (green), and ASPM-1 (red). Bar = 2 μm.
(MOV)

S4 Movie S4 Movie shows a movie stepping through and rotating around an oocyte spindle stained for DNA (blue), microtubules (green), and ASPM-1 (red) following *klp-15/16(RNAi)*. Bar = 2 μm.
(MOV)

S5 Movie S5 Movie shows mitosis in a GFP::histone; GFP::tubulin, *klp-15/16(RNAi)* embryo. Mitotically dividing cell is marked with an asterisk. Bar = 10 μm.
(MOV)

S6 Movie S6 Movie shows anaphase in wild-type (left) and *klp-15/16(RNAi)* (right) oocytes expressing mCherry::histone; GFP::tubulin. Bar = 5 μm (wild-type); 10 μm (*klp-15/16(RNAi)*).
(MOV)

S7 Movie S7 Movie shows anaphase in control (left) and *klp-15/16(RNAi)* (right) oocytes expressing mCherry::histone; SPD-1::GFP and corresponds to Fig 4D. Bar = 5 μm.
(MOV)
