# Supplementary figures and images for: Interplay between microtubule bundling and sorting factors ensures acentriolar spindle stability during C. elegans oocyte meiosis
Source: PLoS Genet. 2017 Sep 14;13(9):e1006986. doi: 10.1371/journal.pgen.1006986 (PMC5614648; doi:10.1371/journal.pgen.1006986)

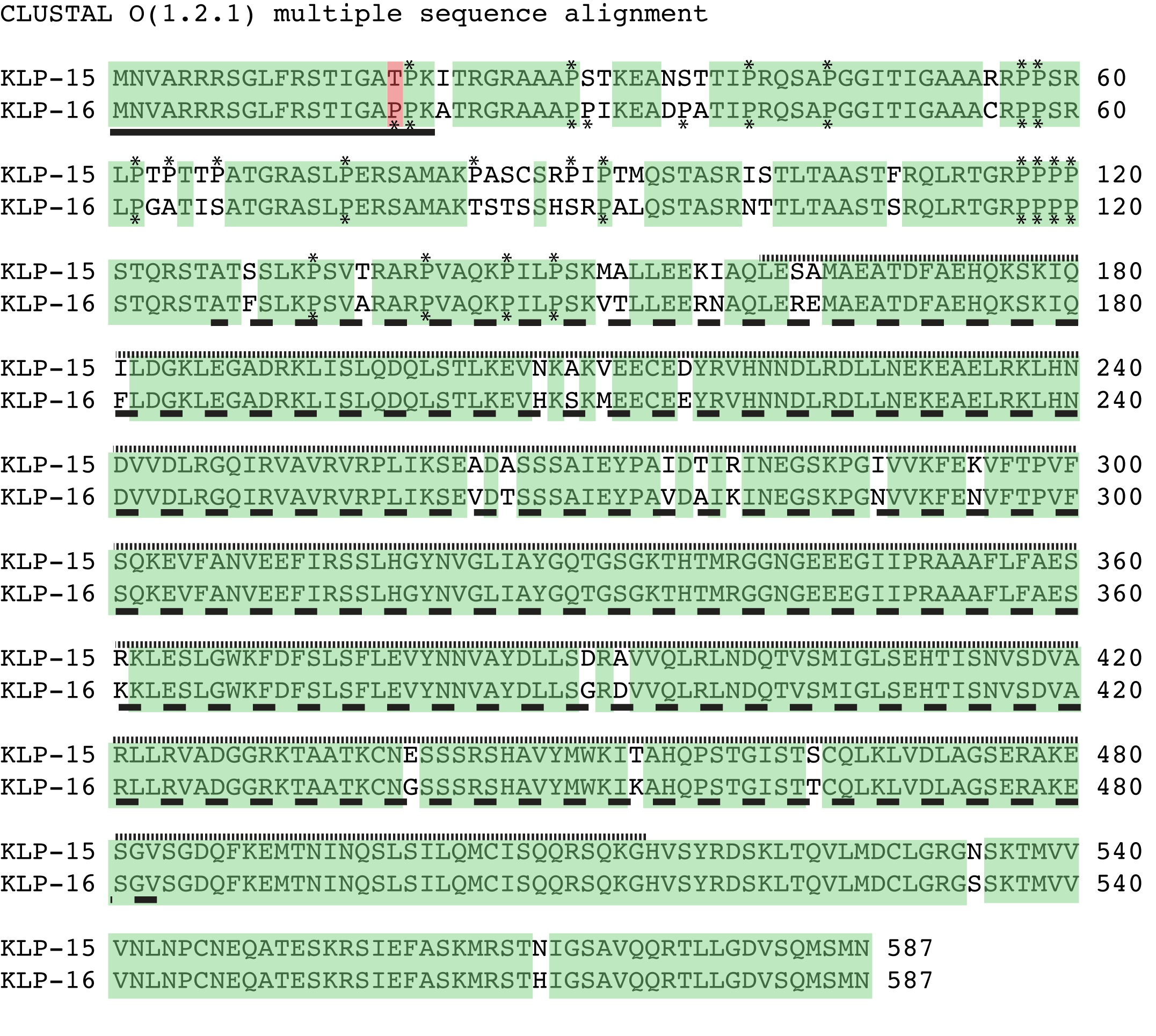

Supplement: S1 Fig — Residues shaded in green are identical between the two proteins. Prolines in the proline-rich tail (1–149 aa) are denoted with asterisks. The dotted line denotes the region of complementarity to klp-15(RNAi) and the dashed line denotes the region of complementarity to klp-16(RNAi). The solid line under the N-terminal 20 amino acids marks the peptide sequence that was used to make our KLP-15/16 antibody; the red shaded residue is the single amino acid difference between KLP-15 and KLP-16 in the sequence used to make the antibody. (TIF) [file pgen.1006986.s001.tif]

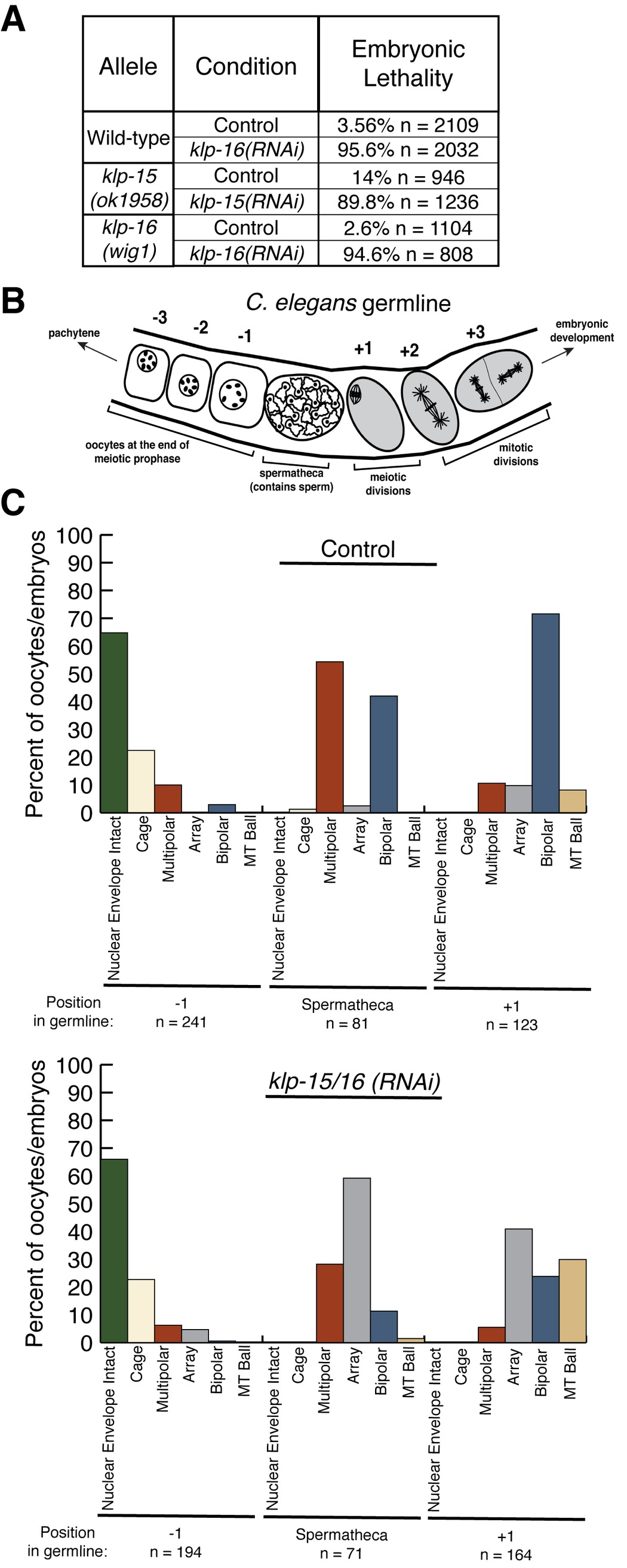

Supplement: S2 Fig — (A) Results of embryonic lethality assays from wild-type, klp-15(ok1958), and klp-16(wig1) worms. The wild-type and klp-16(wig1) worms were fed bacteria expressing the RNAi clone annotated as targeting KLP-16, and klp-15(ok1958) worms were fed bacteria expressing the RNAi clone annotated as targeting KLP-15. (B) Diagram of the C. elegans germline. The germline is organized in an assembly-line fashion where oocytes in prophase (-3 to -1 positions) are ovulated into the spermatheca where they are fertilized. These fertilized embryos begin the meiotic divisions and exit the spermatheca to the +1 position where they continue to progress through meiosis and subsequently mitosis. This organization enables staging of spindles based on the position of the oocyte/embryo in the germline. (C) Quantification of spindle phenotypes in control and klp-15/16(RNAi) worms. This analysis was done using live worms expressing GFP::tubulin, GFP::histone. n represents the number of oocytes/embryos analyzed for each condition. (TIF) [file pgen.1006986.s002.tif]

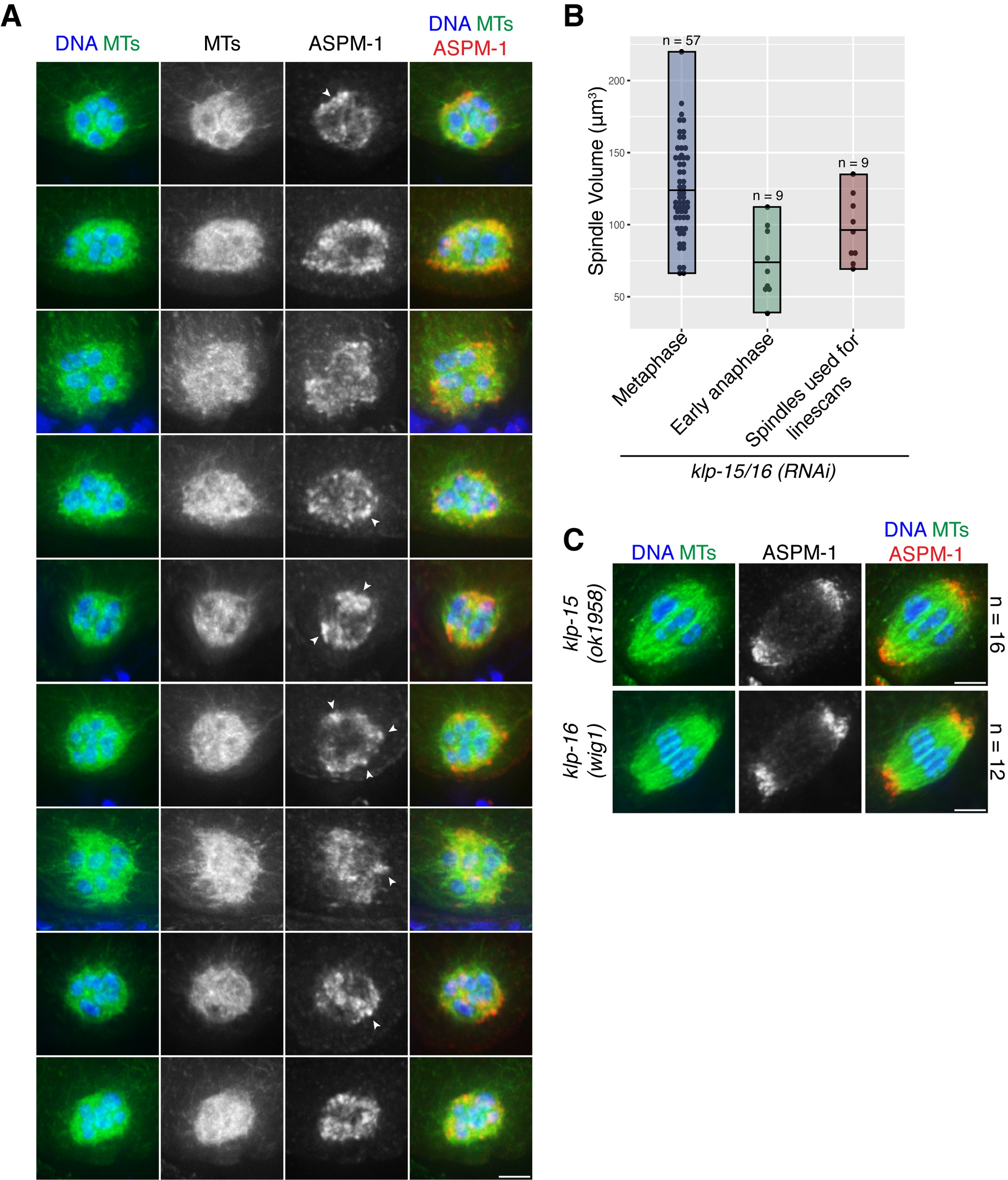

Supplement: S3 Fig — (A) Shown are DNA (blue), microtubules (green), and ASPM-1 (red) for the nine klp-15/16(RNAi) microtubule ball images used for the linescan analysis performed in Fig 1D. ASPM-1 sometimes displays areas of concentration within these structures (examples denoted with arrowheads), that could get averaged out in the graph shown in Fig 1D due to the heterogeneity of the structures. However, we did not observe any clear examples where microtubules appeared to be well-organized into ASPM-1-rich poles that resembled those in wild type spindles, demonstrating that spindle organization is disrupted. (B) Boxplot of spindle volumes of metaphase, early anaphase and the spindles that were used for the linescan analysis in Fig 1D. Metaphase and early anaphase spindles were staged by SEP-1 and AIR-2 localization. Shaded bars represent the range of volumes, bar within the boxes represents the mean, and n represents the number of spindles analyzed for each condition. The range of volumes of the spindles used for linescans suggest that our analysis included both metaphase and early anaphase spindles. (C) DNA (blue), microtubules (green), ASPM-1 (red). In both klp-15(ok1598) and klp-16(wig1) oocytes, spindles are indistinguishable from wild-type spindles. n represents the number of spindles observed for each condition. Bars = 2.5 μm. (TIF) [file pgen.1006986.s003.tif]

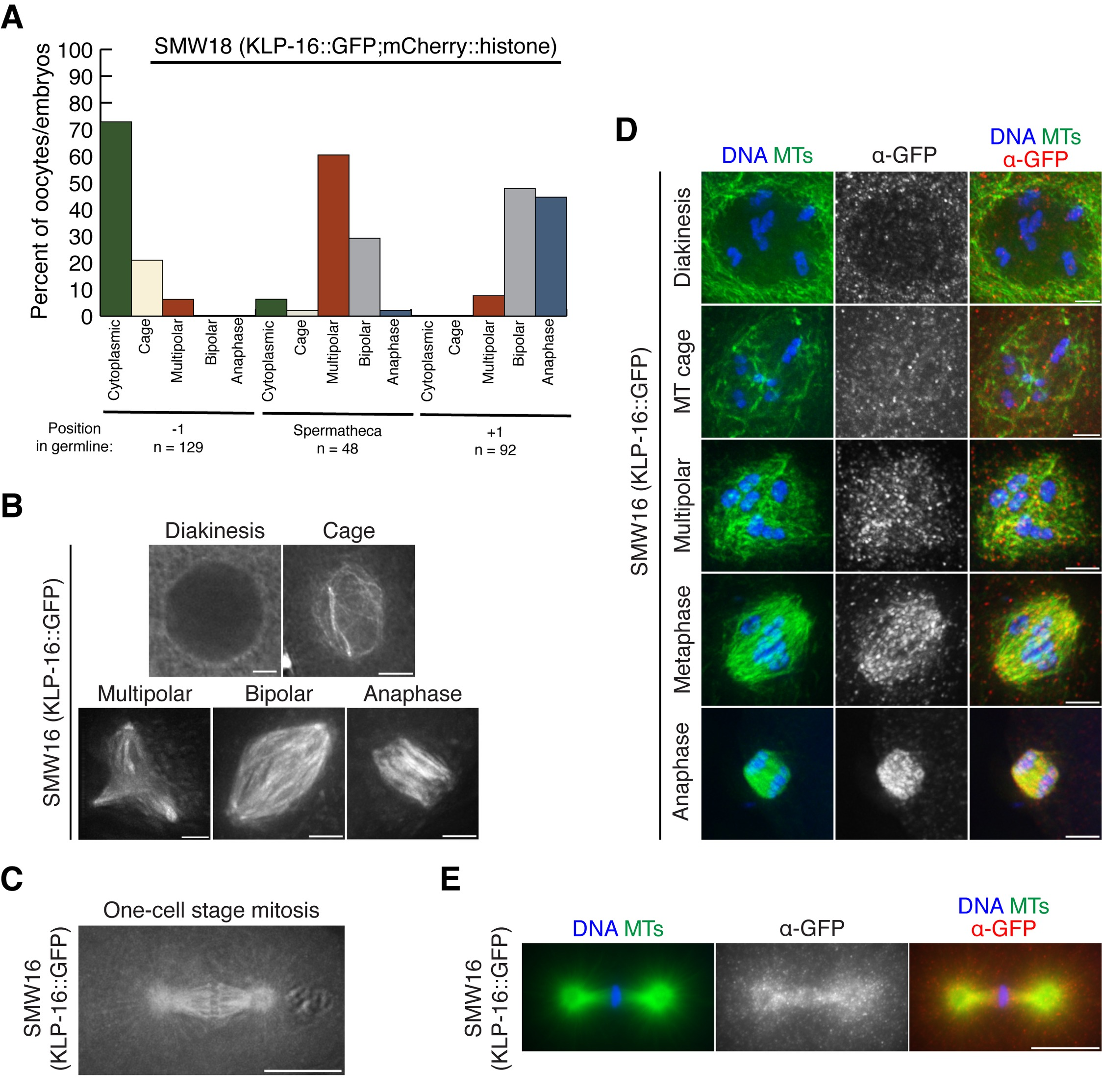

Supplement: S4 Fig — (A) Quantification of KLP-16 localization in live worms expressing KLP-16::GFP and mCherry::histone (SMW18). KLP-16 localization was scored as “cytoplasmic” if the GFP signal was absent from inside the nucleus. At all other stages, the GFP signal was enriched on the spindle near the chromosomes. n represents the number of oocytes/embryos analyzed for each condition. (B and C) Examples of KLP-16 localization in live worms expressing KLP-16::GFP (SMW16). (B) KLP-16::GFP is absent from the nucleus prior to spindle assembly. During spindle assembly, KLP-16 localizes to the spindle microtubule bundles and remains associated through anaphase; the image of the cage stage is a sum projection of the spindle structure. (C) KLP-16 localizes to mitotic spindle microtubules and centrosomes in one-cell stage embryos. (D and E) DNA (blue), microtubules (green), GFP (red). Meiotic (D) and mitotic (E) spindles from worms expressing KLP-16::GFP (SMW16) were stained with an anti-GFP antibody. The staining pattern of KLP-16::GFP on meiotic spindles (D) is identical to the staining pattern using the anti-KLP-15/16 antibody. (E) KLP-16::GFP was detected on spindle microtubules and centrosomes (quantification in Materials and Methods). The mitotic image was not deconvolved. Bars = (B and D) 2.5 μm; (C and E) 10 μm. (TIF) [file pgen.1006986.s004.tif]

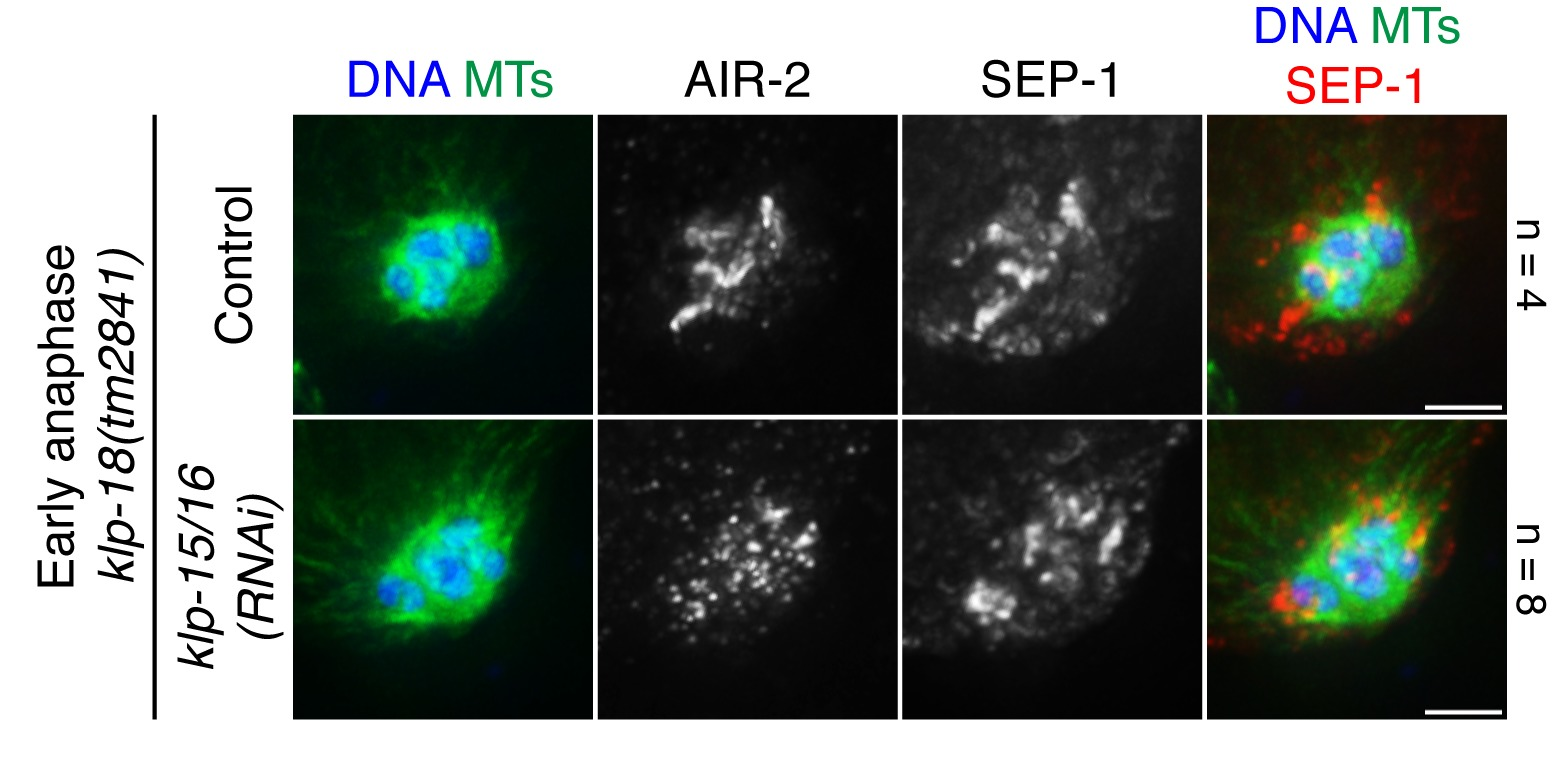

Supplement: S5 Fig — DNA (blue), tubulin (green), AIR-2 (not in merge), and SEP-1 (red in merge). In the klp-18(tm2841) mutant strain, early anaphase spindles (where SEP-1 is colocalized with AIR-2) begin as a ball of microtubules surrounding the chromosomes, in both control and klp-15/16(RNAi) treated oocytes. n represents the number of spindles observed for each condition. (TIF) [file pgen.1006986.s005.tif]
